# A negative modulatory role for rho and rho-associated kinase signaling in delamination of neural crest cells

**DOI:** 10.1186/1749-8104-3-27

**Published:** 2008-10-22

**Authors:** Maya Groysman, Irit Shoval, Chaya Kalcheim

**Affiliations:** 1 Department of Anatomy and Cell Biology, Hebrew University-Hadassah Medical School, Jerusalem 91120, Israel

## Abstract

**Background:**

Neural crest progenitors arise as epithelial cells and then undergo a process of epithelial to mesenchymal transition that precedes the generation of cellular motility and subsequent migration. We aim at understanding the underlying molecular network. Along this line, possible roles of Rho GTPases that act as molecular switches to control a variety of signal transduction pathways remain virtually unexplored, as are putative interactions between Rho proteins and additional known components of this cascade.

**Results:**

We investigated the role of Rho/Rock signaling in neural crest delamination. Active RhoA and RhoB are expressed in the membrane of epithelial progenitors and are downregulated upon delamination. *In vivo *loss-of-function of RhoA or RhoB or of overall Rho signaling by C3 transferase enhanced and/or triggered premature crest delamination yet had no effect on cell specification. Consistently, treatment of explanted neural primordia with membrane-permeable C3 or with the Rock inhibitor Y27632 both accelerated and enhanced crest emigration without affecting cell proliferation. These treatments altered neural crest morphology by reducing stress fibers, focal adhesions and downregulating membrane-bound N-cadherin. Reciprocally, activation of endogenous Rho by lysophosphatidic acid inhibited emigration while enhancing the above. Since delamination is triggered by BMP and requires G1/S transition, we examined their relationship with Rho. Blocking Rho/Rock function rescued crest emigration upon treatment with noggin or with the G1/S inhibitor mimosine. In the latter condition, cells emigrated while arrested at G1. Conversely, BMP4 was unable to rescue cell emigration when endogenous Rho activity was enhanced by lysophosphatidic acid.

**Conclusion:**

Rho-GTPases, through Rock, act downstream of BMP and of G1/S transition to negatively regulate crest delamination by modifying cytoskeleton assembly and intercellular adhesion.

## Background

The neural crest (NC) has long been a model for understanding cell migrations during development [1-5]. Nonetheless, the molecular network underlying the generation of cellular movement remains incompletely understood [6,7]. This process involves an epithelial-to-mesenchymal transition (EMT) of the premigratory NC cells residing in the dorsal neural tube (NT) followed by delamination.

Bone morphogenetic protein (BMP), Wnt and fibroblast growth factor (FGF) signals were implicated in NC specification and lineage segregation [8-13] and evidence illustrates the involvement of BMP and Wnt in subsequent NC delamination and/or migration [14-20]. Our studies showed that a balance between BMP and its inhibitor noggin underlies the emigration of trunk-level NC independently of earlier cell specification [20]. A decreasing rostrocaudal gradient of BMP4 activity is established along the NT by a reciprocal gradient of noggin. Noggin downregulation is, in turn, triggered by the developing somites, which thus determine the timing of NC emigration [18-20]. BMP then induces EMT of NC by triggering Wnt1 transcription. The latter promotes G1/S transition, which is a necessary step for delamination of trunk NC [14,21].

Acting downstream of BMP and/or Wnt, transcription factors such as Sox9, FoxD3 and Slug were found to be sufficient for inducing some properties of NC differentiation when ectopically expressed in the neuroepithelium [22-24]. Moreover, when expressed in combination they induced some features of EMT [24] whereas when separately provided they were unable to promote NC delamination in the trunk [23-26]. Successful delamination also requires the activity of effector genes that act on re-organization of the actin cytoskeleton, alterations in adhesive properties and consequent loss of epithelial polarity [13,27-30]. In this context, N-cadherin was found to be a component of the BMP-dependent network leading to NC EMT. N-cadherin inhibits the onset of NC delamination both by a cell adhesion-dependent mechanism as well as by repressing canonical Wnt signaling. Relief from N-cadherin-mediated inhibition is attained in the dorsal NT during the onset of cell emigration. This is accounted for by an ADAM10-dependent cleavage of the full-length molecule into a soluble domain with pro-delamination properties, a process triggered by BMP [31].

Additional candidates for orchestrating NC delamination include the RhoGTPases. RhoGTPases are molecular switches that control a variety of signal transduction pathways; they are known primarily for their pivotal role in regulating the actin cytoskeleton, but not less significant are their effects on cell polarity, gene transcription, G1 cell cycle progression, membrane transport, and so on (reviewed in [32-34]). Rho proteins cycle between two conformational states, one bound to GTP, the active form, and the other bound to GDP, the inactive state. This switch is regulated by several activators and inactivators [35,36]. The role of Rho GTPases in NC development remains virtually unexplored. Association between ectoderm and intermediate neural plate, a paradigm known to induce NC features [37,38], activates transcription of Rho-related proteins [39]. Transcription of *rhoA *and *rhoB*, but not of *rhoC*, was detected in the avian NT with *rhoB *being expressed in premigratory and early migrating NC [40]. Initial *rhoB *mRNA is induced by BMP signaling [40], which is also required for its continuous transcription [20]. In contrast, Wnt3a appears to have no effect on the onset of *rhoB *expression [41], yet Wnt6 was sufficient to stimulate prematurely both *rhoA *and *rhoB *mRNAs [42]. Furthermore, inhibition of either canonical or non-canonical Wnt signaling had no effect on maintenance of *rhoB *in the dorsal NT [14]. In contrast to avians, in mouse and *Xenopus*, *rhoB *mRNA is expressed in migrating NC but not in the NT [43,44]; another, unconventional RhoGTPase termed RhoV was found instead to be expressed in the early *Xenopus *NC and to be required for its specification [45]. The first functional study performed in avians *in vitro *suggested that Rho activity was required for NC delamination but not for subsequent cell migration [40]. However, loss of RhoB function in mice exhibited no apparent morphogenetic defects, although the development of the NC was not directly monitored [46]. Furthermore, in different cellular contexts, Rho signaling has been shown to promote cell migration or, conversely, to maintain the epithelial state [47-49]. Based on the above, it was important to investigate its function in the NC, a *bona fide *model for generation of cellular movement, and to begin understanding how it integrates within the already known molecular network leading to NC delamination.

Here we show that Rho signaling, through Rho-associated kinase (Rock) activity, negatively modulates the onset of NC emigration both in explants and *in vivo*. *In vivo *loss-of-function of either RhoA or RhoB enhanced emigration of NC cells. Likewise, inhibiting Rho signaling by C3 transferase stimulated the process. Consistently, treatment of explanted neural primordia with membrane-permeable C3 or with the Rock inhibitor Y27632 both accelerated and enhanced NC emigration. None of the above affected NC cell proliferation. Furthermore, they altered crest morphology by reducing stress fibers and focal adhesions. They also caused premature downregulation of membrane-associated N-cadherin. Reciprocally, activation of endogenous Rho by lysophosphatidic acid (LPA) inhibited emigration while enhancing stress fibers, focal adhesions and N-cadherin. The effect of LPA was specific to Rho as Y27632 rescued the observed phenotypes. Since NC delamination is triggered by BMP and depends upon successful G1/S transition, we examined possible interactions between these pathways. Blocking Rho or Rock rescued NC delamination in explants treated with noggin or with a G1/S inhibitor. In the latter case, G1-arrested cells successfully emigrated. Reciprocally, BMP4 was unable to rescue cell emigration upon inhibition with LPA. Together, our findings suggest that Rho-GTPases, acting through Rock, negatively regulate NC delamination by modifying cytoskeleton assembly and cell-cell adhesions and acting downstream of BMP and of G1/S transition.

## Results

### Loss of the membrane-bound, active form of RhoA and RhoB is associated with the EMT of NC cells

**Figure 1:**
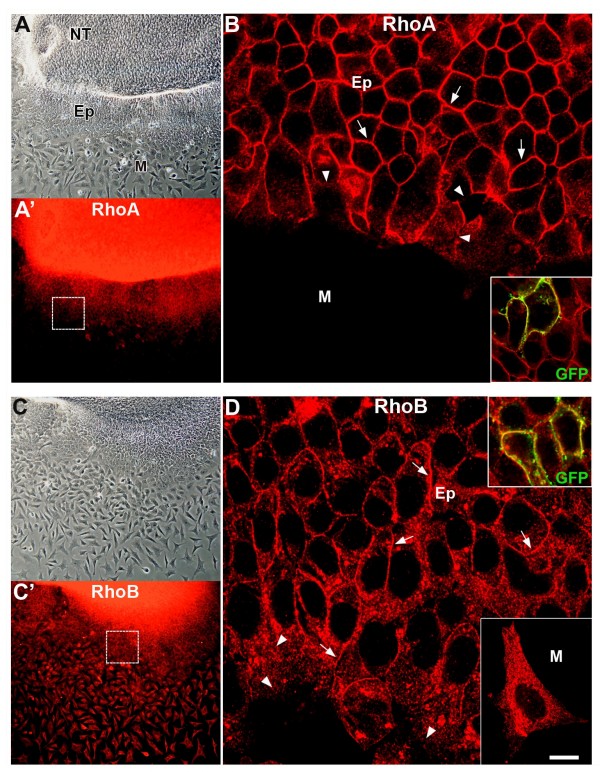
**Localization of RhoA and RhoB immunoreactive proteins to explanted neural primordia.** (A,B) RhoA (SC418) is expressed in the neural tube (NT) and in the adjacent flattening epithelioid sheet (Ep) but is downregulated in mesenchymal (M) NC cells. (A) Phase contrast; (A') RhoA; (B) higher magnification of the inset in (A') showing a membrane-associated pattern (arrows) that progressively disappears from cells about to delaminate (arrowheads) until full downregulation in mesenchymal progenitors. (C,D) RhoB (SC180) is expressed in the NT, in the epithelioid sheet and in mesenchymal NC cells. (C) Phase contrast; (C') RhoB; (D) higher magnification of the inset in (C') showing both membrane-associated RhoB (arrows), which disappears from cells about to delaminate (arrowheads), and a cytoplasmic staining that is kept in the mesenchymal cells (M, inset). Insets in (B,D) show expression of a GFP variant following electroporation that associates with the surface membrane (green). Note yellow staining reflecting colocalization with RhoA or RhoB. Bar: 22 μM (A,A',C,C'); 5 μM (B,D) and insets; 3.5 μM, lower inset in (D).

**Figure 2:**
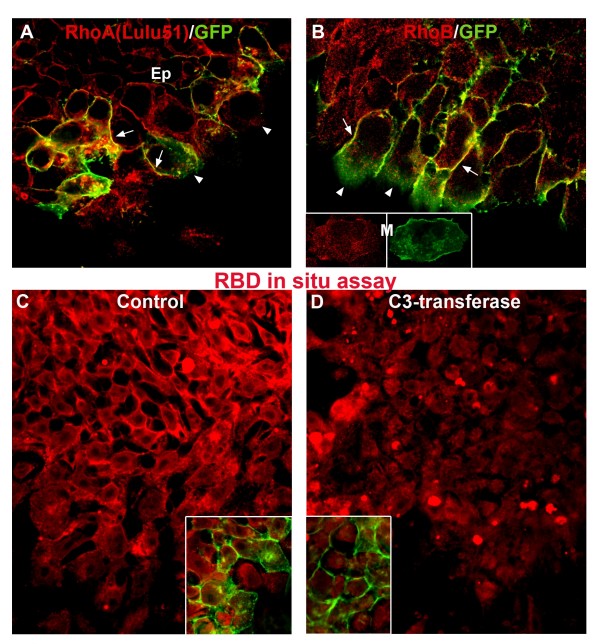
**Active Rho is membrane-associated.** (A) RhoA (Lulu51) and (B) monoclonal anti-RhoB (see Results) are apparent in the membrane of epithelial progenitors (arrows) where they co-localize with membrane-GFP, and progressively disappear from cells about to delaminate (arrowheads). Insets in (B) show cytoplasmic staining in a mesenchymal cell (M) but absence of membranous immunoreactivity when compared to GFP. All patterns are similar to that observed with the antibodies depicted in Figure 1. (C,D) *In situ *assay with the Rho-binding domain of Rothekin (RBD-GST). Note the membranal pattern apparent in epithelial cells co-localizing with membrane GFP (C, yellow in inset), and absence of cell surface pattern in explants treated with C3 transferase (D, and also inset showing green fluorescence of GFP but not of RBD-GST in the membranes). Bar = 7.5 μM.

*RhoA *and *rhoB *transcripts are present in the dorsal NT at stages corresponding to the production and emigraton of NC cells [20,40]. Here we characterize the expression of RhoA and RhoB immunoreactive proteins to NTs explanted for 20 h. RhoA was recognized using two specific antibodies (SC418 and Lulu51) and so was RhoB (SC180 and a monoclonal anti-RhoB [40]), and similar expression patterns were observed for each antibody pair. RhoA and RhoB were apparent in the neuroepithelium and in the flattening epithelioid sheet (Figures 1 and 2). In these, immunostaining was membrane-associated (Figures 1B,D and 2A,B), as confirmed by colocalization with a membrane-linked form of yellow fluorescent protein (YFP) in cells following electroporation (insets in Figures 1B,D and 2A,B). The latter pattern characterizes the active forms of Rho proteins [34] (and see below). Upon delamination, mesenchymal NC cells totally downregulated membrane-bound Rho proteins. A progressive disappearance was already detectable in the front of cells about to delaminate (Figures 1B,D and 2A,B, arrowheads). In addition, RhoB exhibited a cytoplasmic distribution that was maintained in the emigrated mesenchymal NC (Figure 1C, Figure 1D, inset, and Figure 2B, inset).

To further confirm that the membrane-associated staining reflects active Rho proteins, explants were incubated with the Rho-binding domain of Rhotekin fused to glutathione S-transferase (RBD-GST), which specifically recognizes the GTP-bound form of Rho proteins, followed by indirect immunofluorescent detection with an antibody to GST. Consistent with the expression data described above, epithelial progenitors exhibited active Rho proteins in a membrane-associated pattern that colocalized with membranous green fluorescent protein (GFP), and this surface staining disappeared upon cell dissociation (Figure 2C and inset, and data not shown). To control for the specificity of this reaction, explants were pretreated with C3 transferase, which inhibits ADP-ribosylation of all Rho proteins [50] but not of Cdc42 or Rac [51]. No membranous staining was detected under these conditions (Figure 2D and inset). Taken together, these results suggest that Rho signaling is active in the epithelial NC progenitors prior to EMT.

### Inhibition of Rho/Rock activities promotes premature and enhanced NC delamination

**Figure 3:**
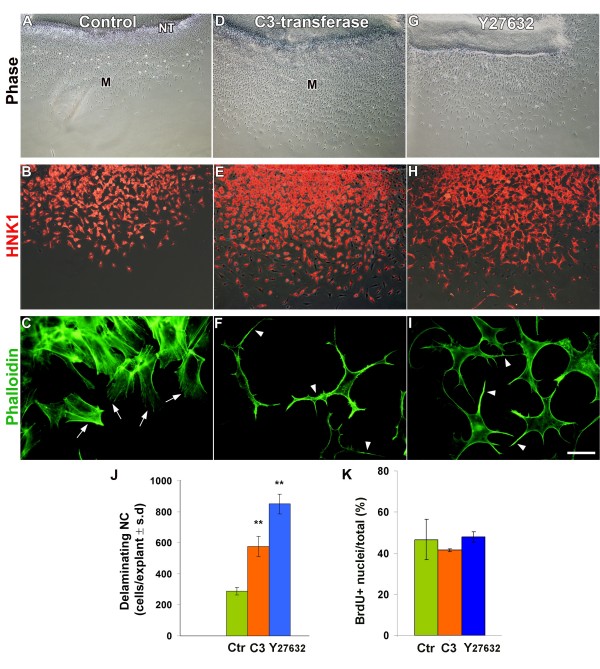
**Inhibition of Rho/Rock activities enhances neural crest (NC) delamination from explanted neural primordia.** (A,D,G) Phase contrast. (B,E,H) HNK-1. (C,F,I) Phalloidin staining of F-actin. In control explants (A,B), NC delamination is progressive, transiting through an epithelioid stage on the way to a mesenchymal phenotype. (B,E,H) Delaminating cells are HNK-1+ under all experimental conditions. (C,F,I) Control delaminating cells have F-actin+ stress fibers (arrows in (C)), whereas C3- and Y27632-treated explants show cortical actin but lack stress fibers. Instead, they adopt irregular morphologies with long protrusions (arrowheads in (F,I)). (J) Both C3 transferase and Y27632 stimulate NC delamination when compared to control (Ctr) (**p < 0.001) and promote the direct production of mesenchymal cells without epithelioid intermediates (D,E,G,H), but have no significant effect on cell proliferation (K). Bar: 100 μM, (A,D,G); 36 μM (B,E,H); 8 μM, C,F,I. BrdU, bromo-deoxyuridine; M, mesenchymal; NT, neural tube; SD, standard deviation.

To begin examining the possible effects of Rho and Rock signaling [52] on NC delamination, their activities were inhibited in explants and *in ovo*. Rho GTPase activity was selectively blocked through the use of C3 transferase (see preceding section). Notably, C3 exotoxin only slightly penetrates intact cells; thus, high concentrations were required for activity (50–200 μg/ml [40]) which might compromise some cellular functions. To overcome this limitation, we used a new membrane-permeable version of the enzyme [53,54] that enabled us to lower by 50- to 200-fold the concentration of drug (to 1 μg/ml) while still keeping its expected biological activity (that is, disruption of actin stress fibers and altered cellular morphology; Figure 3C,F). At 16 h in the presence of C3 transferase, a 2.4-fold increase in the number of delaminating NC cells was monitored over control values (N = 5 counted cultures out of 12 showing a similar phenotype; Figure 3J).

Likewise, the specific Rock inhibitor Y27632 [55-57] was similarly applied to neural primordia excized from the level opposite the unsegmented mesoderm. At 16 h of incubation, the number of cells present on the substrate of Y27632-treated cultures was 3.2-fold higher compared to that monitored under control conditions (N = 6 counted cultures for control and Y27632 out of 10 and 16 showing a similar phenotype, respectively' Figure 3J). Notably, NC delamination in the presence of the Rock inhibitor was already evident as early as 2 h after drug addition when compared to control tubes in which the first delaminating cells became apparent about 9 h following explantation (Additional file 1A,C), indicating that premature delamination is not accounted for by increased cell proliferation. Cells delaminating in the presence of either C3 or Y27632, like their control counterparts, expressed HNK-1, confirming their NC identity (Figure 3B,E,H). In contrast to controls, however, they directly transited into a mesenchymal phenotype not exhibiting the characteristic dense intermediate epithelioid conformation (Figure 3A,D,G; Additional file 1B,C,D). Consequent to both treatments, the phenotype of cells was dramatically altered, with remarkable stellate morphologies and many long protrusions. This was accompanied by, and likely to result from, a loss of F-actin+ stress fibers (Figure 3C,F,I; Additional files 2 and 3). In spite of enhancing cell emigration, the proportion of proliferating cells was not increased upon treatment with either C3 or Y27632 (Figure 3K, N = 5 for each treatment). Hence, inhibition of Rho/Rock signaling in explants both enhances and accelerates delamination of NC progenitors while disrupting the F-actin cytoskeleton but without affecting their proliferation.

**Figure 4:**
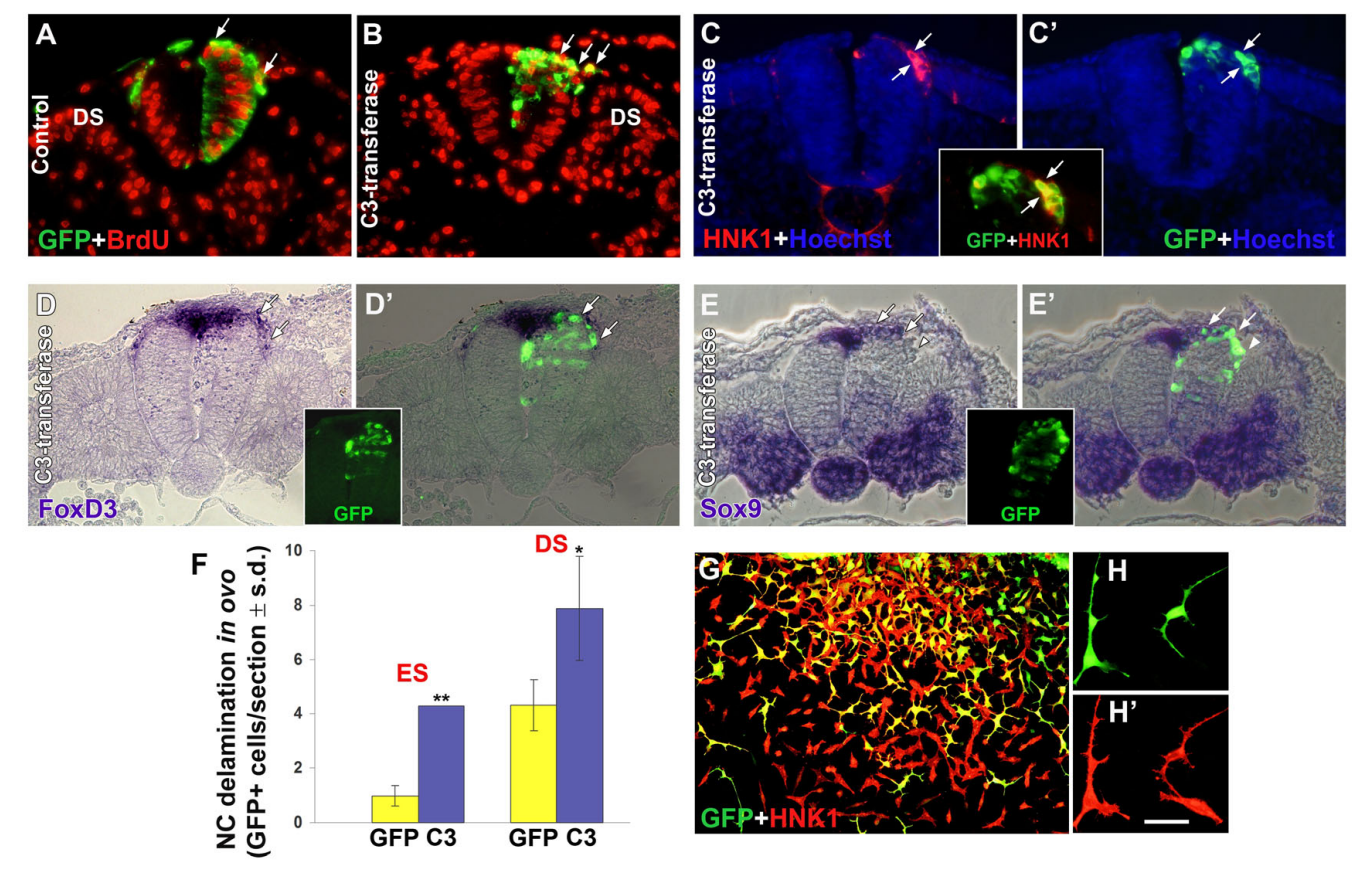
**Inhibition of Rho activity by C3-DNA stimulates neural crest (NC) delamination *in ovo*.** (A-E) Transverse sections of embryos that received control green fluorescent protein (GFP) (A) or C3-DNA/GFP (B-E). Most delaminating cells contain bromo-deoxyuridine (BrdU)+ nuclei (arrows in (A,B)). (C,C' and inset) Electroporation of C3/GFP followed by HNK-1 labeling. (D,D'and inset) Electroporation of C3/GFP followed by *in situ *hybridization with *FoxD3*. (E,E'and inset) Electroporation of C3/GFP followed by *in situ *hybridization with *Sox9*. Note that delaminating, C3-containing cells (green) co-express all NC markers (arrows). Note also in (E,E') that the front of ventrally migrating precursors (arrowhead) is C3/GFP+ but has downregulated *Sox9*, which is normally transcribed in the dorsal neural tube and rapidly lost from emigrating NC. (F) Quantification of NC delamination at epithelial somite (ES) and dissociating somite (DS) levels. Electroporation of C3-transferase into hemi-NTs enhanced NC delamination opposite both epithelial somite and dissociating somite levels (*p < 0.05, **p < 0.01 compared to GFP-control). (G,H,H') C3-transfected cells co-express HNK-1. Neural primordia electroporated with C3/GFP were explanted. Delaminating NC cells co-express GFP and HNK-1 and exhibit a characteristic change in morphology. Bar: 42 μM (A-E); 50 μM (G); 20 μM (H,H').

To further examine whether inhibition of Rho activity similarly affects NC delamination *in ovo*, C3-DNA and GFP-DNA were co-electroporated into hemi-NTs opposite the segmental plate and the extent of NC delamination was monitored 16 h later at epithelial and dissociating somite levels. A clear stimulation of delamination of GFP+ cells was measured at both segmental levels (4.4-fold and 1.8-fold, respectively; Figure 4A,B,F, and data not shown) when compared to control GFP-treated embryos. As previously reported [14,21,31], most GFP+ delaminating cells were bromo-deoxyuridine (BrdU)+ and so were the delaminating progenitors that received C3-transferase (Figure 4A,B), showing that inhibition of Rho signaling has no adverse effect on G1/S transition (shown in later figures and data not shown). To ascertain their NC identity, C3/GFP-treated embryos were co-stained with HNK-1 (Figure 4C,C') or *in situ *hybridized with *FoxD3 *(Figure 4D,D') and *Sox9 *(Figure 4E,E'). In all cases, GFP+ delaminating cells co-expressed the three markers (arrows) yet *Sox9 *was consistently downregulated from the front of the ventrally migrating GFP+ progenitors, as it primarily marks the premigratory NC (Figure 4E,E', arrowhead). C3 also caused a mild dissociation of neuroepithelial progenitors ventral to the NC domain, yet these did not contribute to the NC migratory pathways. To further verify that transfected C3-DNA was active, neural primordia were electroporated *in ovo *and then explanted. C3/GFP-positive cells vigorously delaminated, co-expressed HNK-1 further confirming their NC identity, and adopted irregular morphologies with multiple and long processes similar to those observed upon treatment with either soluble C3 or Y27632 (Figures 3 and 4G,H,H').

**Figure 5:**
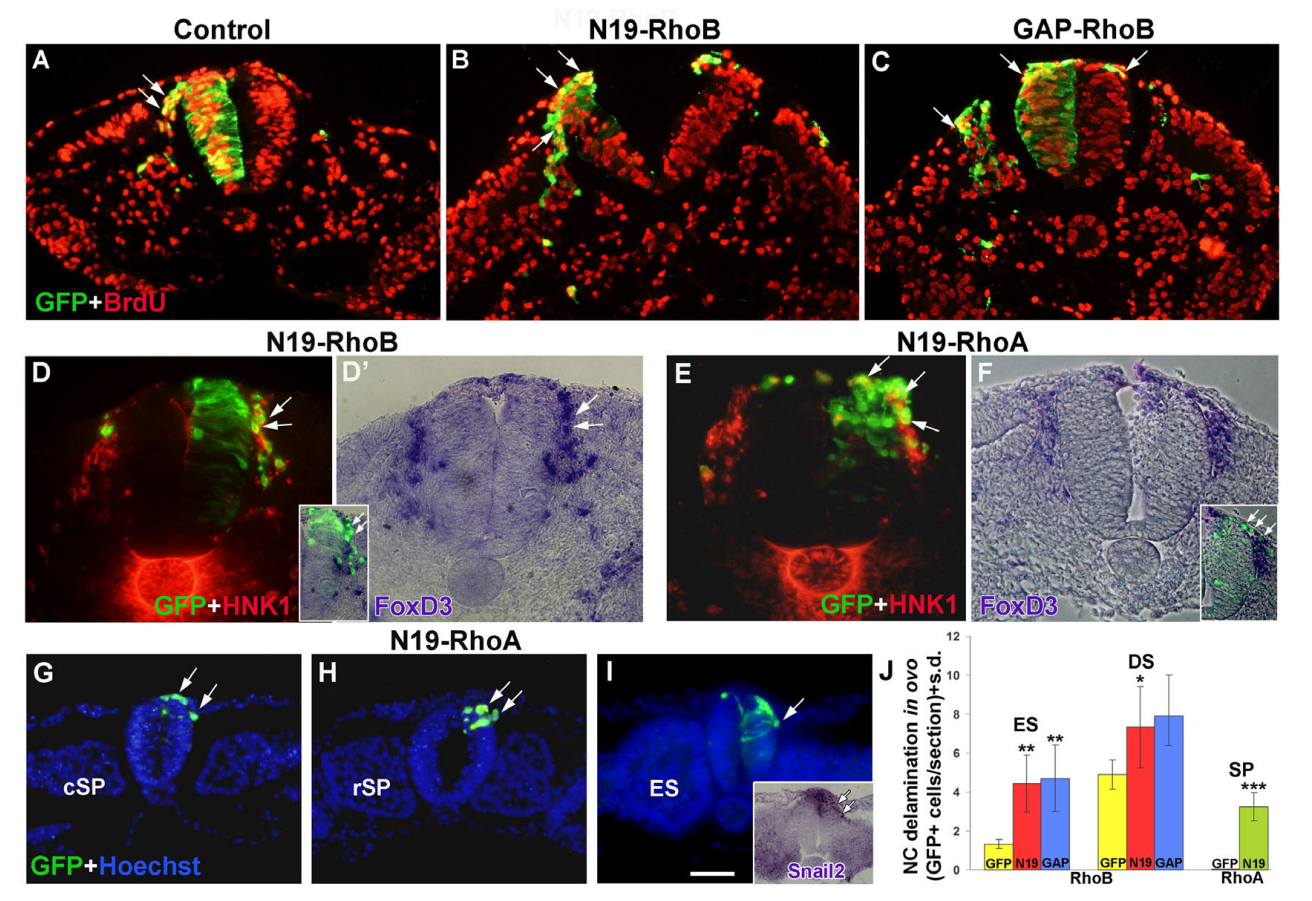
**Inhibition of either RhoA or RhoB activities enhances neural crest (NC) delamination *in ovo***. (A-C) Transverse sections at a dissociated somite level showing the emigration of control green fluorescent protein (GFP)-expressing (A,A'), N19-RhoB-expressing (B,B') and GAP-RhoB-expressing (C,C') NC cells (green). Note in (A',B',C') that most delaminating progenitors are also bromo-deoxyuridine (BrdU)+ (red; arrows pointing at double-labeled cells). (D,D') Transverse section at a similar level as in (A-C) of a hemi-NT that received N19-RhoB (green) and was further stained for HNK-1 (red in (D)) and *FoXD3 *mRNA (D' and inset). Transfected emigrating cells are positive both for HNK-1 and *FoXD3 *(arrows). (E, F) Two transverse sections of embryos that received N19-RhoA (green) and were further processed for HNK-1 immunostaining (E, red) or *FoXD3 in situ *hybridization (F and inset). Note that delaminating cells that received N19-RhoA also express HNK-1 or *FoxD3 *(arrows). Note as well that the transfected hemi-NT cells lost their normal pseudostratified appearance and are rounded. (G-I) Transverse sections through the caudal segmental plate (cSP) level (G), rostral segmental plate (rSP) level (H), and recently formed epithelial somite (ES) (I) of embryos that received N19-RhoA. Note premature delamination of NC progenitors expressing N19-rhoA/GFP (arrows) on a background of Hoechst nuclear stain (blue) or co-expressing *Snail2 *mRNA (inset in (I)). No delamination from these axial levels is observed upon transfection of control GFP (not shown). Note that at these very caudal levels of the axis, electroporation was predominantly dorsal due to positioning of the electrodes at a slightly more rostral level in order not to damage the gastrulating area of the axis. (J) Quantification of NC delamination (*p < 0.05, **p < 0.01, ***p = 0.0001). DS, dissociating somite; ES, epithelial somite; s.d., standard deviation; SP, segmental plate. Bar: 45 μM (A-F); 53 μM (G-I); 83 μM, insets in (D,E,I).

Since C3 transferase blocks activity of all Rho proteins, we next monitored the *in vivo *effects of inhibiting either RhoA or RhoB separately. Inhibition was achieved by overexpression of N19-RhoA or N19-RhoB, which lack GTPase activity [58], or by the chimeric construct GAP-rhoB. The latter was prepared by fusing the RhoGAP domain of p190, a GTPase activating protein that accelerates intrinsic GTPase activity, with the carboxy-terminal hypervariable sequence of RhoB, which confers specificity to individual Rho proteins [59]. First, we monitored their effects on the F-actin cytoskeleton. To this end, neural primordia were electroporated and then explanted. Whereas control-GFP-treated cells contained stress fibers as well as cortical actin in their periphery, NC cells that received mutant Rho constructs were devoid of stress fibers when compared to their untransfected neighbors and to control-GFP (Additional file 2, and data not shown). Next, we examined their effects on NC delamination *in ovo*. Both N19-RhoB and GAP-RhoB enhanced NC delamination opposite both epithelial (3.4- and 3.6-fold over control, respectively) and dissociated somites (1.5- and 1.6-fold over control, respectively) (Figure 5A–C,J, and data not shown). As with C3 transferase (Figure 4), the effects were more pronounced at earlier stages when fewer control cells emigrated. Since the same embryos were analyzed for both axial levels, values monitored adjacent to epithelial somites stemmed from electroporations that attained the caudal segmental plate level. We assume, therefore, that the delay between transfection and onset of emigration, which is longest at this level, enabled a more efficient expression of the plasmids prior to the beginning of cell emigration and, hence, led to a greater effect. In addition, the delaminating GFP+ NC progenitors were also BrdU+ (Figure 5A,B,C, arrows), further extending the results obtained with C3 and altogether demonstrating that loss of Rho function has no adverse effect on G1-S transition. The identity of delaminating N19-RhoB/GFP+ progenitors was additionally assessed by co-staining with HNK-1 and *in situ *hybridization with *FoxD3*. The N19-RhoB/GFP+ emigrating cells co-expressed both HNK-1 and *FoxD3 *markers, substantiating their NC identity (Figure 5D,D' and inset).

In contrast to both constructs that inhibited RhoB activity, treatment with N19-RhoA caused a dissociation of neuroepithelial cells when examined after 16 h, consistent with its broader expression pattern in the NT [40]. This confirms the relative specificities of the constructs used to either RhoA or RhoB. In spite of hemi-NT dissociation (note round cells in Figure 5E, F compared to pseudostratified progenitors in N19-RhoB in Figure 5D), the emigrating N19-RhoA/GFP+ progenitors co-expressed both HNK-1 and *FoxD3*, confirming they are NC cells and also suggesting that labeled central nervous system progenitors that dissociated did not contribute to the NC migratory pathway. Likewise, no central progenitors were found to join the NC migratory pathways upon treatment with C3 transferase (Figure 4), altogether suggesting that dissociation of central progenitors is not sufficient for inducing their migration. Because of its adverse effect on NT progenitors, counting the number of emigrating cells in N19RhoA-treated embryos at advanced stages was less compelling; therefore, electroporations were performed for a shorter period at a very caudal level of the axis and embryos were fixed 8–10 h later, corresponding to the levels of the caudal or rostral segmental plate or early epithelial somite, respectively. In these regions, control-GFP+ NC cells were still confined to the NT, with no measurable cell delamination (data not shown). In contrast, premature NC delamination occured in neural primordia that received N19-RhoA while transfected central nervous system progenitors were still pseudostratified (Figure 5G–J). Furthermore, the early delaminating cells co-expressed NC-specific markers such as *Snail2 *(Figure 5I, inset), *FoxD3 *(see later figure) and *Sox9 *(not shown). No such effect was observed when transfecting either N19-RhoB or GAP-RhoB, further substantiating the specificity of the tools employed.

Taken together, our data demonstrate that loss of Rho function both *in vivo *and in explants facilitates the onset of NC emigration, suggesting that endogenous Rho plays a negative role in the process. The observation that both enhanced and accelerated delamination also occurs when inhibiting Rock further suggests that Rho acts via Rock signaling to maintain NC cells in an epithelial state.

### Activation of Rho signaling with lysophosphatidic acid inhibits the onset of NC emigration

**Figure 6:**
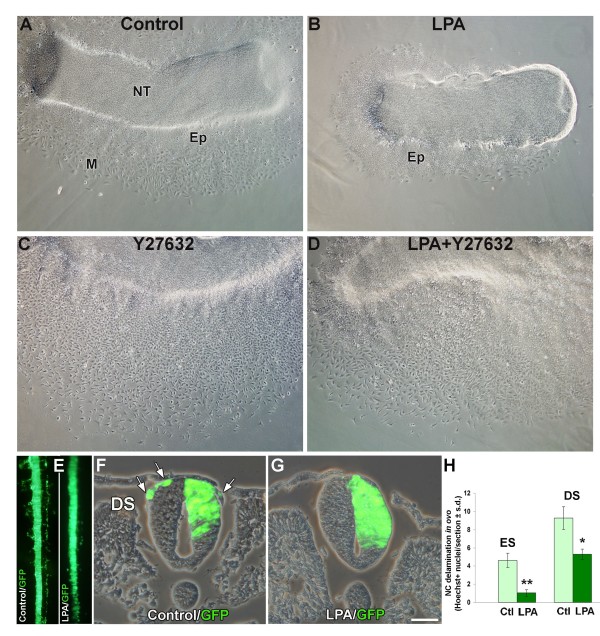
**Lysophosphatidic acid (LPA)-mediated activation of Rho signaling inhibits neural crest (NC) delamination in explants and *in ovo***. (A,B) Treatment of explanted neural primordia with LPA enables flatterning of the epithelioid sheet but virtually inhibits generation of mesenchymal (M) NC (B) when compared to controls (A). (C,D) Y27632 alone promotes vigorous cell delamination (C) and reverts LPA-induced inhibition (D). (E,F,G) Application of control gel (E, left, and F) or of LPA mixed with pluronic gel (E, right and G) dorsal to neural tubes (NTs) *in ovo *was preceded by unilateral electroporation of green fluorescent protein (GFP)-DNA. (E) Dorsal view of two living embryos. (F,G) Transverse sections. Note emigration of labeled NC cells in (E, left panel) and F (arrows) and lack of emigrating cells in (E, right panel) and (G). (H) Application of LPA mixed with pluronic gel dorsal to NTs reduces NC delamination *in ovo *opposite both epithelial (ES) and dissociating somites (DS) (*p < 0.05, **p < 0.01). Ctl, control gel. Bar: 70 μM (A-D); 25 μM (F,G).

To further investigate whether Rho/Rock activity negatively modulates NC delamination, we adopted a gain of function approach and overexpressed full-length and constitutively active forms of RhoA, RhoB, Rock1 and Rock2 DNAs *in vivo *by electroporation. Both GTPases as well as Rock proteins caused the death of the transfected cells, precluding further analysis [24,60]. To circumvent this limitation, endogenous Rho activity was stimulated by treatment with LPA. LPA is a bioactive phospholipid that signals through G-protein-coupled serpentine receptors and, in different cell types, it promotes cytoskeletal reorganization through activation of the Rho pathway [51,61-63]. Treatment of neural primordia with 1 μg/ml LPA enabled the flattening of epithelial cells on the substrate but virtually prevented delamination of NC cells in all cases examined (N = 12) when compared to untreated controls (N = 12) (Figure 6A,B). These flattening progenitors were HNK-1+ (data not shown) yet retained N-cadherin (see below, Interactions between Rho/Rock, N-cadherin and the actin cytoskeleton underlie NC delamination), thus representing prospective NC cells prior to EMT. To control whether the effect of LPA was accounted for by inhibiting Rho signaling through Rock, explants were simultaneously treated with LPA and Y27632. NC delamination was then rescued in all explants examined (Figure 6C,D; N = 16) and similar results were obtained when co-treating LPA with C3 (not shown).

Next, small pieces of LPA-containing pluronic gel or of control gel were grafted dorsal to the NT *in ovo *and the number of Hoechst+ NC cells apparent dorsal to the neural primordium and up to the dorsomedial border of the somites was monitored. A bilateral decrease in the extent of NC emigration was observed opposite both epithelial and dissociating somite levels in NTs that received LPA over controls (77.2% and 42.6%, respectively; N = 4 for each treatment; Figure 6H). No apparent difference in cell survival was observed between the above treatments (data not shown). To further visualize this effect, control GFP was electroporated unilaterally into NTs immediately before application of the pluronic gel. As shown in Figure 6E–G, labeled cells emigrated from control neural primordia, but not from tubes that received LPA. Hence, stimulation of endogenous Rho function inhibits NC delamination *in vivo *and in explants, in further support of a negative regulatory role of Rho proteins.

### Rho-GTPase activity does not affect specification to the NC lineage

**Figure 7:**
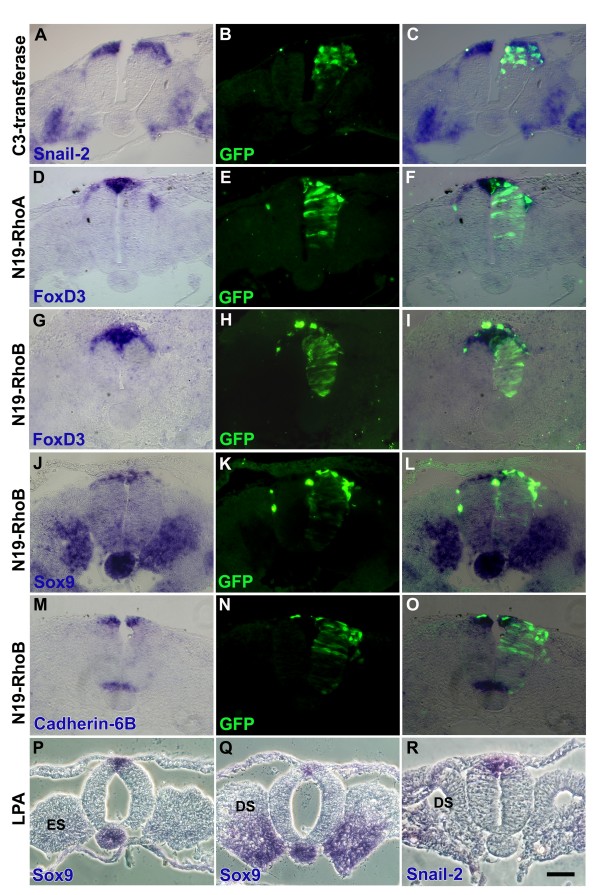
**Loss or gain of Rho function has no effect on early expression of neural crest (NC)-specific genes.** (A-C) Electroporation of C3/green fluorescent protein (GFP; green) followed by *in situ *hybridization for *Snail2 *(blue). (D-F) Electroporation of N19-RhoA/GFP (green) followed by *in situ *hybridization for *FoxD3 *(blue). (G-O) Electroporation of N19-RhoB/GFP (green) followed by *in situ *hybridization for *FoxD3 *(G-I), *Sox9 *(J-L) or *Cad6B *(M-O). *Sox9 *and *Cadherin-6B *are primarily expressed in the premigratory NC, hence in (L,O) most of the transfected progenitors undegoing early delamination (green) are unlabeled for the above genes. (P-R) Lysophosphatidic acid (LPA)/pluronic gel placed on top of the dorsal portion of embryos and further processing for *Sox9 in situ *hybridization at epithelial and dissociating somite levels (ES and DS, respectively) and for *Snail2 *(R). Note that unilateral electroporation did not change the patterns of gene expression in the dorsal neural tube (NT) when compared to the contralateral sides or to control GFP embryos (not shown). Likewise, treatment with LPA did not alter either early gene expression or further maintenance despite inhibiting NC cell emigration. Bar: 45 μM.

Our data suggest that the observed effects of Rho gain and loss of function on NC emigration cannot be explained by changes in cell proliferation or survival. Therefore, we examined whether enhanced delamination upon Rho loss of function can be accounted for by recruitment of ventral neuroepithelial cells to the putative NC pool or, conversely, by loss of NC properties in the LPA-treated embryos. To test for these possibilities, embryos were treated with C3 transferase, N19-RhoA, N-19-RhoB or LPA at the segmental plate level of the axis and *in situ *hybridized 8 h later to visualize the expression patterns of early NC-specific markers, such as *Snail2*, *Sox9*, *FoxD3 *and *Cadherin 6B*. No ventral expansion of the domain of any of the above genes was observed when Rho activity was abrogated compared to the contralateral intact side (Figure 7A–O). Reciprocally, no loss of marker expression in the dorsal NT could be seen upon LPA treatment when examined at epithelial or even dissociating somite levels, in spite of the bilateral inhibition of cell delamination (Figure 7P–R). Altogether, these results suggest that Rho activity does not affect the specification of epithelial progenitors to the NC fate (Figure 7) or the subsequent maintenance of their identity (Figures 4 and 5).

### Interactions between Rho/Rock, N-cadherin and the actin cytoskeleton underlie NC delamination

To examine the mechanism by which Rho signaling negatively affects NC delamination, explants were stained for F-actin, N-cadherin and vinculin. While epithelial cells in control explants exhibited a dense array of stress fibers, the delaminating NC cells expressed significantly fewer yet they retained cortical actin bundles circumscribing the cell periphery. Treatment with Y27632 that stimulated NC delamination caused a total loss of stress fibers and a significant change in cell morphology (Additional file 3A,B; Figure 3). Reciprocally, LPA-induced inhibition of NC EMT was associated with a dramatic increase in stress fiber density and both effects were reversed by co-treatment with Y27632 (Additional file 3C,D).

**Figure 8:**
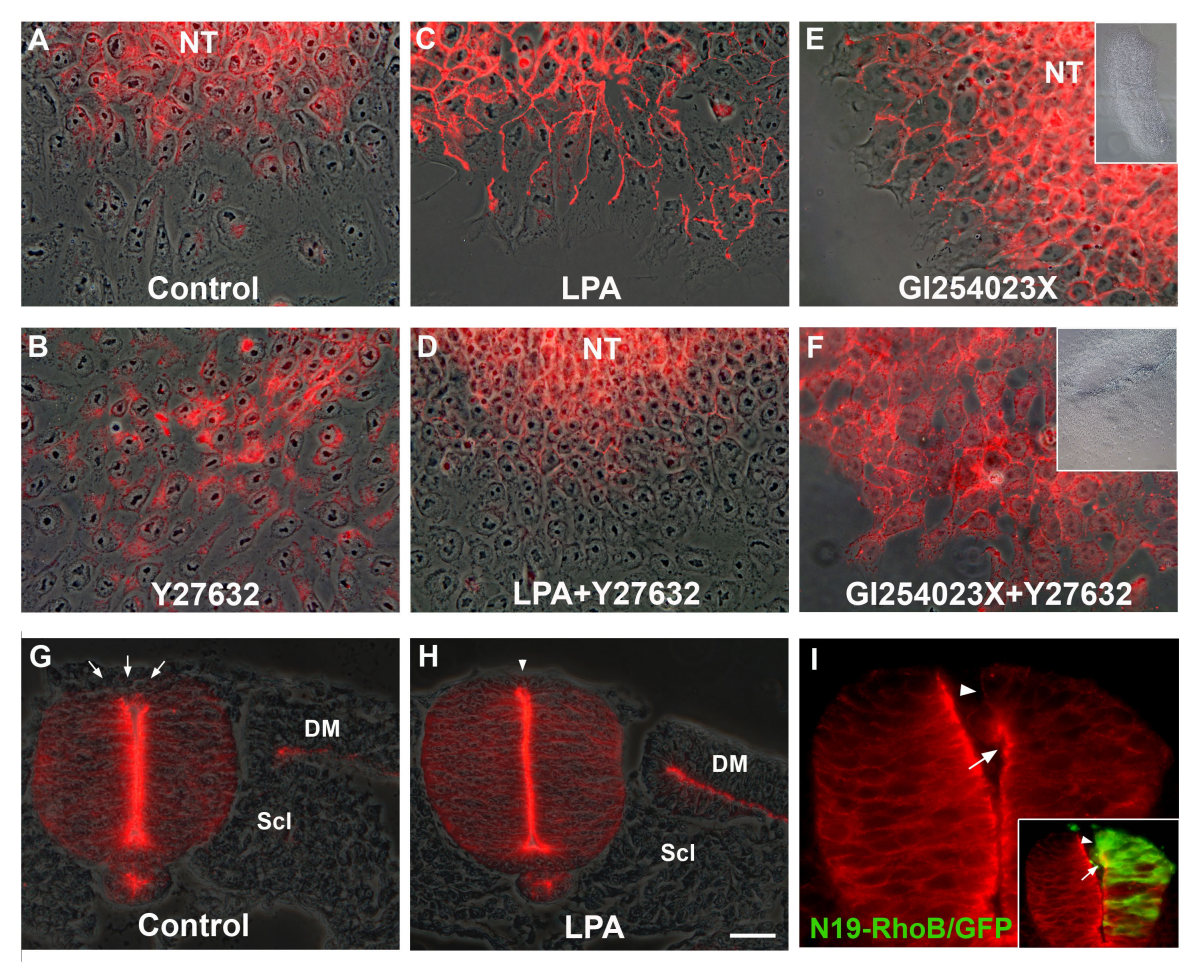
**Rho/Rock signaling stabilize membrane-bound N-cadherin while inhibiting neural crest (NC) delamination.** (A) In control explants, N-cadherin (red) is expressed in the membrane of epithelial and epithelioid cells flattening onto the substrate but is downregulated upon delamination in mesenchymal cells. (B) N-cadherin disappears from the membrane of Y27632-treated cells that exhibit a mesenchymal phenotype even close to the neural tube (NT). (C) Lysophosphatidic acid (LPA) strenghtens membranous N-cadherin immunoreactivity while inhibiting epithelial-to-mesenchymal transition (EMT). (D) These effects of LPA are reverted by co-treatment with Y27632. (E,F) Inhibition of N-cadherin cleavage by GI254023X keeps membrane-associated N-cadherin and prevents NC EMT; both effects are reverted by inhibiting Rock activity. Note in (F) that membrane N-cadherin staining is either fragmentary or absent. Insets show low magnification of phase contrast images. In all panels, the NT explant is to the top. (G,H) Treatment with LPA/pluronic gel *in vivo *prevents the normal downregulation of N-cadherin from adherens junctions (arrowhead in (H)) that is seen in control gel-treated embryos (arrows in (G)) at dissociated levels of the axis. (I and inset) Loss of RhoB function by transfection of N19-RhoB/green fluorescent protein (GFP) prematurely downregulates N-cadherin from adherens junctions in the apical NT when compared to the untreated side (arrowhead pointing at the apical side of the NT expressing N19/GFP but devoid of N-cadherin). Note that electroporation at more ventral domains of the NT was without effect on N-cadherin staining (ventral to the arrow), consistent with the dorsally restricted expression of RhoB. DM, dermomyotome; Scl, sclerotome. Bar: 15 μM (A-F); 500 μM (insets in E,F); 26 μM (G,H); 14 μM (I); 65 μM, inset in (I).

**Figure 9:**
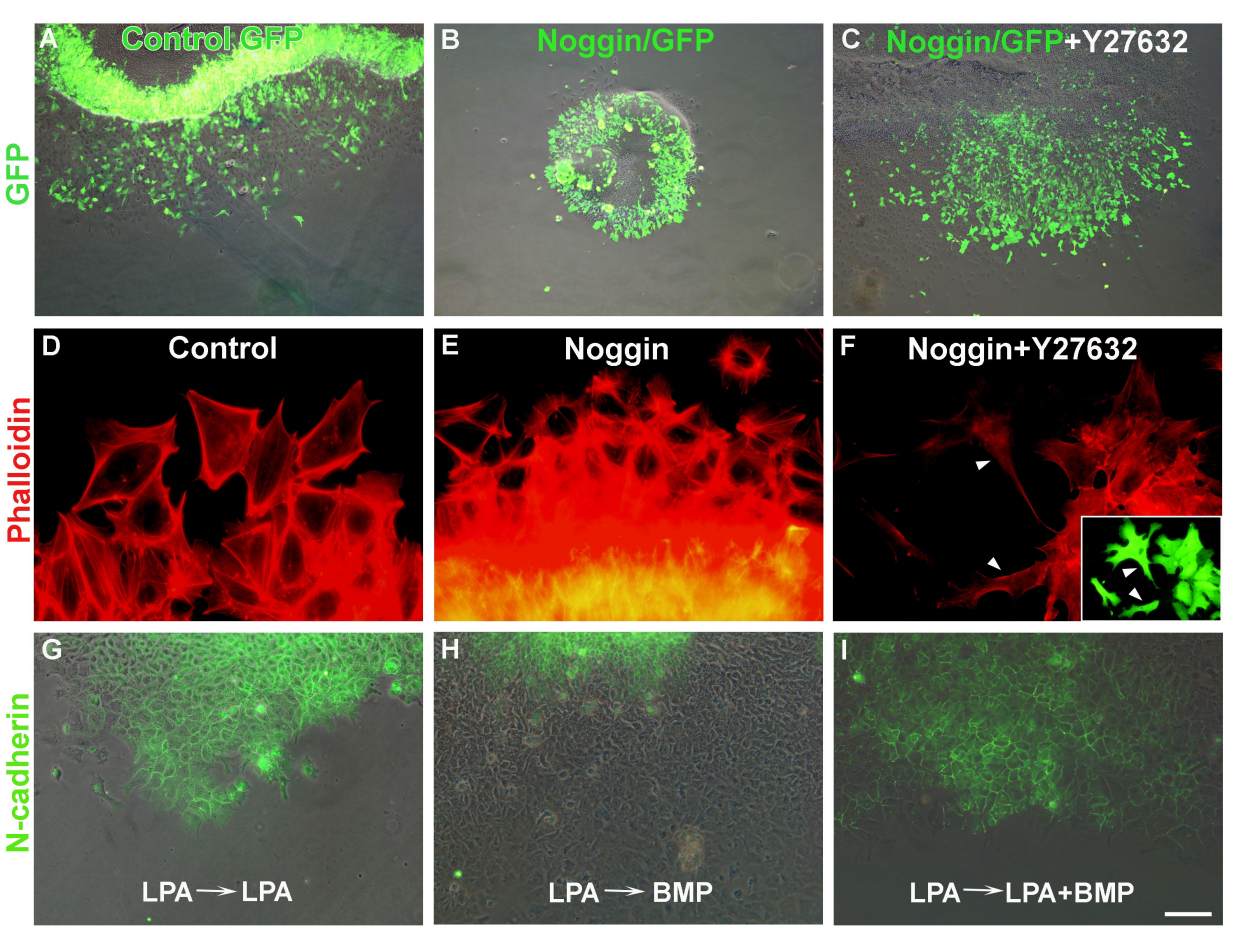
**Rock signaling acts downstream of bone morphogenetic protein (BMP)/noggin.** (A-F) Neural primordia explanted after *in ovo *electroporation with control green fluorescent protein (GFP) (A,D), noggin/GFP (B,E), or noggin/GFP and Y27632 (C,F). While noggin inhibited delamination and augmented formation of stress fibers, Y27632 rescued both processes. (A-C) Merged GFP immunofluorescence and phase contrast. (D-F) Phalloidin staining showing enhancement or loss of stress fibers upon noggin or noggin+Y27632 treatments, respectively. Inset in (F) shows morphology of transfected cells (arrowheads). (G-I) Treatment of NT explants with lysophosphatidic acid (LPA) prevents neural crest (NC) delamination and maintains membrane-bound N-cadherin (green) (G). Replacing LPA by BMP4 reverts cell delamination and downregulates N-cadherin (H). In contrast, in the continuous presence of LPA, BMP4 is unable to rescue NC emigration or N-cadherin loss from the cell membranes (I). Bar: 80 μM (A-C); 4 μM (D-F); 8 μM, inset in (F); 42 μM (G-I).

Previously, we reported that inhibition of ADAM 10-dependent cleavage of N-cadherin with GI254023X, which maintains the full-length protein in a membrane-bound conformation, prevented NC delamination [31]. Here we show that treatment with GI254023X also results in a stable cytoskeleton rich in F-actin stress fibers, similar to the phenotype of LPA-treated NTs. Importantly, application of Y27632 reversed both GI254023X-induced effects (Additional file 3E,F). These observations underscore an interaction between N-cadherin and Rho/Rock via regulation of F-actin dynamics. To directly explore this issue, we stained neural primordia for N-cadherin. In control explants, N-cadherin protein was strongly expressed in the membrane of epithelial progenitors as well as in the epithelioid cells flattening on the substrate. N-cadherin immunoreactivity was, however, lost from delaminating cells that adopted a mesenchymal phenotype (Figure 8A) [31]. In contrast, in the presence of Y27632, the cells adjacent to the NTs were already devoid of membrane-associated N-cadherin and appeared separated from each other, suggesting they lost intercellular adhesions prematurely (Figure 8B; see also Figure 3 and Additional file 1). Consistent with this observation, electroporation of N19-RhoB or C3 resulted in rapid downregulation of N-cadherin protein from adherens junctions in the transfected dorsal hemi-NT *in ovo *(Figure 8I, arrowhead), yet had no effect further ventrally where endogenous RhoB is absent (Figure 8I, arrow and ventralward, and data not shown). Reciprocally, LPA maintained and even upregulated N-cadherin membrane expression whereas co-treatment with Y27632 rescued NC delamination and also reduced N-cadherin immunoreactivity (Figures 8C,D and 9G). Consistent with the explant data, *in ovo *treatment with LPA, which inhibited NC emigration (Figure 6), maintained N-cadherin associated with adherens junctions in the dorsal NT at axial levels where N-cadherin has been normally downregulated (Figure 8G,H) [31]. Likewise, treatment with GI254023X, which inhibited NC emigration, maintained membrane-associated N-cadherin in explants; when co-treatment with Y27632 was performed, membranous N-cadherin was either completely lost or fragmentary and NC cells underwent EMT (Figure 8E,F).

Next, we examined the presence of focal contacts by immunostaining for vinculin. Control delaminating cells exhibited vinculin-containing focal attachment points. While treatment with Y27632 markedly reduced their number, LPA stimulated them (Additional file 4). This indicates that the number of cell-substrate contacts depends on Rho/Rock activity and is inversely related to the extent of NC emigration. Together, these results suggest that stimulation or inhibition of NC delamination by Y27632 or LPA, respectively, is associated with, and likely to operate via, regulation of cell adhesion and cytoskeletal properties. Therefore, to delaminate successfully, epithelial NC cells need to downregulate Rho/Rock activities, which, in turn, diminish the number of stress fibers and abrogate N-cadherin-mediated adhesion.

### Rho/Rock signaling acts downstream of BMP/noggin in the regulation of NC delamination

Downregulation of *noggin *in the dorsal NT relieves BMP activity from the inhibition to which it is subjected along the caudal NT [20]. BMP4 then triggers NC delamination via the canonical Wnt pathway [14]. Moreover, N-cadherin, which inhibits NC delamination, is part of this molecular cascade [31]. Since we show that Rho activity negatively modulates NC delamination and also alters N-cadherin expression, we asked whether Rho/Rock signaling are part of the BMP-dependent network leading to NC emigration.

Electroporation of noggin-DNA along with GFP-DNA into hemi-NTs *in ovo *inhibited delamination of NC cells following explantation of the neural primordia when compared to control epithelia in which GFP+ NC cells normally delaminated (Figure 9A,B; N = 12 for each treatment). This was accompanied by an increase in stress fiber density similar to that observed in LPA and GI254023X-treated explants (Figure 9D,E). To examine whether this was caused by an increase in Rho/Rock activity, noggin-transfected hemi-NTs were treated with Y27632, which dramatically reduced the amount of stress fibers and rescued NC delamination (Figure 9C,F; N = 16). Similar results were obtained upon treatment with C3 transferase (data not shown).

Next, we examined the ability of BMP4 to rescue cell delamination in explants in which the process was inhibited by LPA. As previously described, continuous treatment with LPA for 16 h (with one change to fresh LPA 8 h after explantation) virtually prevented NC delamination and the maintenance of epitheliality was confirmed by positive staining of membranous N-cadherin (Figure 9G; N = 12). When LPA was washed away and replaced by BMP4, cell emigration with concomitant loss of N-cadherin immunoreactivity was apparent within a few hours (Figure 9H; N = 7). In contrast, when LPA was replaced by a mixture of LPA and BMP4, the latter was unable to rescue cell delamination and the pattern of N-cadherin very much resembled that of LPA only-treated explants (Figure 9I; N = 14). These results suggest that Rho/Rock act downstream of BMP/noggin in the dorsal NT.

### Inhibition of Rho/Rock activity rescues delamination of G1-arrested NC cells

**Figure 10:**
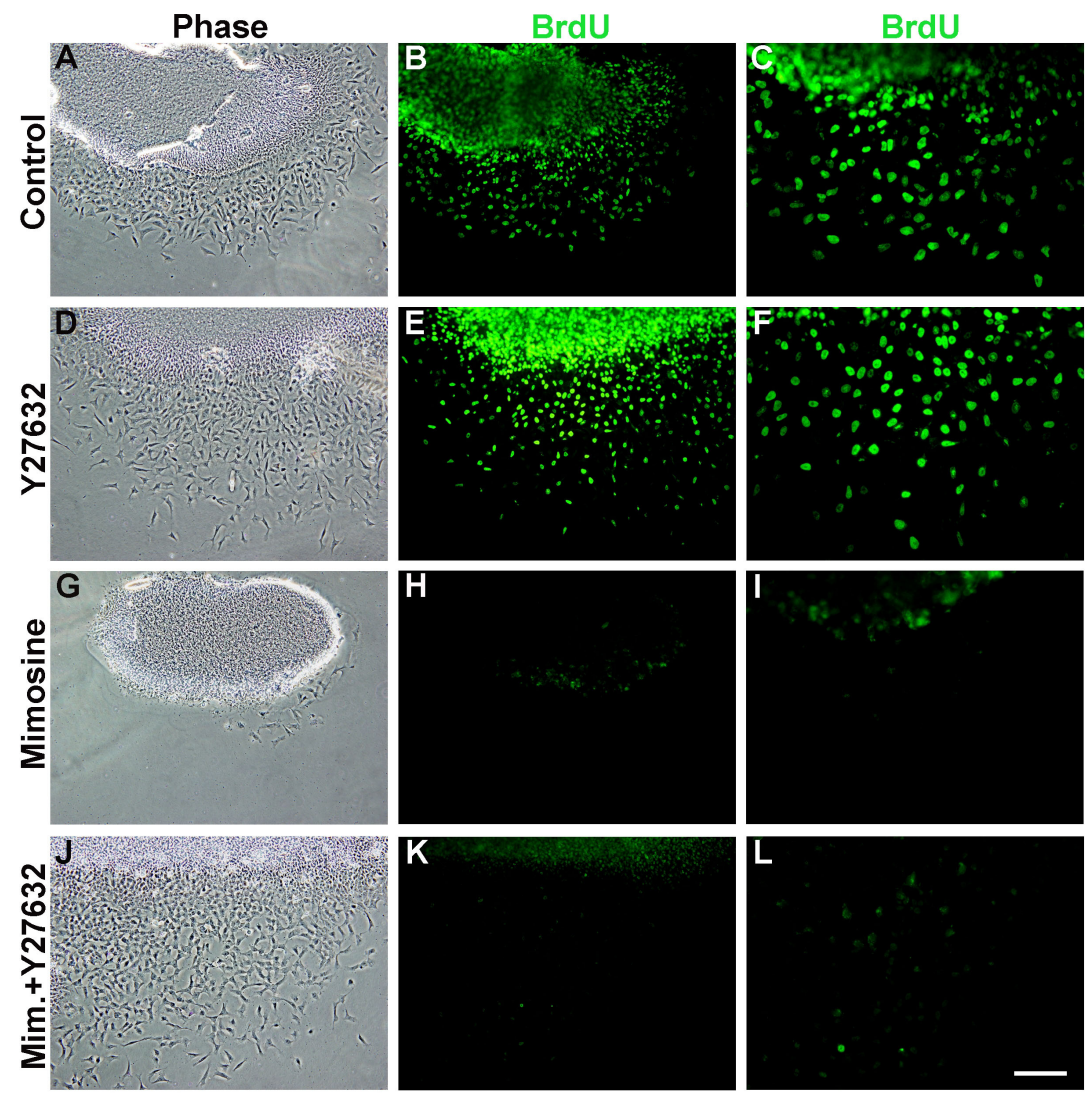
**Inhibition of Rock activity rescues delamination of G1-arrested neural crest (NC) cells**. (A,D,G,J) Phase contrast. (B,C,E,F,H,I,K,L) Bromo-deoxyuridine (BrdU) immunostaining. (C,F,I,L) Higher magnifications to appreciate the front of the delaminated NC cells. Inhibition of Rock with Y27632 enhanced delamination of BrdU+ NC cells ((D-F) compared to (A-C)). Mimosine blocked both BrdU incorporation and NC delamination following 9 h (G-I), whereas cotreatment with mimosine (Mim) and Y-27632 rescued emigration of G1-arrested, BrdU-negative NC cells (J-L). Bar: 45 μM (A,B,D,E,G,H,J,K); 24 μM (C,F,I,L).

We previously showed that trunk NC cells delaminate in the S-phase of the cell cycle and that G1-S transition is a prerequisite for cell emigration [21]. Furthermore, we demonstrated that BMP acts through canonical Wnt signaling that stimulates G1/S transition and NC delamination [14]. To further investigate the relationship between Rho/Rock activity and the BMP-dependent cascade, we prevented G1/S transition with the plant amino-acid mimosine, which induces expression of p27, and asked whether inhibition of Rock activity by Y27632 would rescue G1/S transition and NC emigration. As previously shown [21], mimosine (600 μM) completely blocked both BrdU incorporation and NC delamination from neural primordia explanted for 9 h, the approximate length of one cell cycle (Figure 10A–C,G–I; N = 16). Treatment with Y27632 facilitated delamination of NC cells and had no adverse effect on BrdU incorporation, with a proportion of BrdU+ cells similar to that in controls (Figure 10A–F, and data not shown; N = 16). Notably, co-treatment with mimosine and Y27632 rescued delamination of NC cells, which subsequently dispersed from the explanted epithelium, but the emigrating cells were BrdU-negative, indicating they were still arrested at G1 (Figure 10J–L; N = 16). A similar picture was obtained when explants were treated with membrane-soluble C3 and mimosine (data not shown), further suggesting that Rho proteins via Rock act downstream of G1/S transition to modulate NC emigration.

## Discussion

Growing evidence illustrates that EMT of NC cells followed by cell delamination are modulated by a complex network of interacting transcription factors, cell adhesion molecules and other regulatory proteins. A more complete knowledge of the component genes and their mechanisms of action is required for understanding such a basic developmental mechanism. A role for Rho signaling in this context was highly expected yet remained unclear. We report that Rho/Rock signaling helps maintain premigratory cells in an epithelial state. This is supported by several lines of evidence. The C3 exoenzyme is commonly used as an inhibitor of ADP ribosylation of Rho proteins, thus selectively preventing their activity [50,51]. Addition of membrane-permeable C3, which inhibited the formation of actin stress fibers, resulted in enhanced NC emigration from explanted NTs and so did C3-encoding DNA when missexpressed *in ovo*. In contrast, previous data stemming from NT explants showed that soluble C3 inhibited NC delamination, hence implicating Rho proteins, in particular, RhoB, as a positive signal in the process [40]. However, relatively high levels of the transferase were used in that study, perhaps to overcome its poor penetrance into intact cells. We reason that such high concentrations might have compromised some cellular functions related to the process of interest. Instead, recent availability of a membrane-permeable version of the enzyme enabled us to lower by 50- to 200-fold the concentration of drug applied to similar explants while still keeping its expected biological activity. To challenge our results by independent means, we overexpressed two specific dominant-negative forms of RhoB that also enhanced NC delamination. Furthermore, N19-RhoA stimulated premature cell emigration. Moreover, inhibition of Rock activity both advanced and enhanced the process dramatically. Reciprocally, stimulation of Rho activity by LPA prevented NC delamination. Furthermore, inhibition of NC delamination achieved with either LPA or the ADAM10 inhibitor GI254023X, the latter preventing N-cadherin degradation, was reversed by the Rock inhibitor Y27632 or by C3 (Figures 6 and 8; Additional file 3; MG and CK, unpublished results). Altogether, our loss and gain of function analyses, performed *in ovo *and in explants, demonstrate that Rho proteins act as negative signals in the process of EMT and delamination. This is consistent with our observaton that active Rho proteins are present in the epithelial progenitors and are lost from dissociating cells. It is important to stress that in different biological systems, Rho proteins were shown to act distinctly. Whereas in several instances Rho/Rock mediate the maintenance of the epithelial state, consistent with our data (see, for example, [49,64]), in others, like in untransformed colon epithelial cells, Rho proteins maintain adherens junctions and epitheliality yet exert this effect via Dia rather than Rock, which instead disrupted intercellular adhesions [65]. Furthermore, during endocardial cushion development, inhibition of Rock blocked EMT [66] rather than stimulating the process as observed in the NC. Missexpression of RhoA in post-otic NC cells was reported to alter patterns of NC migration even if virtually no active RhoA was detected in the intact migrating cells. Yet, RhoA missexpression had only a minor effect on cell delamination [67]; the level of active RhoA was, however, not assessed in the premigratory NC. These differences between post-otic and trunk NC are consistent with increasing data sustaining that cranial NC progenitors emigrate from the neuroepithelium as groups rather than individually and, therefore, use different mechanisms than those documented for the trunk [7,68]. These and other results suggest that the multiplicity of activities mediated by Rho proteins and their downstream effectors should be carefully considered in a context-dependent fashion.

Which of the Rho proteins is active in this biological context? The two Rho proteins present in the avian NT are RhoA and RhoB; in contrast, RhoC was undetectable [40] (MG and CK, unpublished observations). Our functional assays show that loss of RhoA as well as of RhoB function enhanced emigration of NC cells *in ovo*. Consistent with previously published specificity data (see Results), we confirm that the dominant negative constructs used are selective as they had distinct effects on the integrity of the neuroepithelium. N19-RhoA caused a dissociation of the hemi-NTs, consistent with the broader expression of *RhoA *mRNA in the NT when compared to *RhoB *[40], whereas neither N19-RhoB nor GAP-RhoB altered general neuroepithelial morphology. In addition, N19-RhoA, but not N19-RhoB, caused a premature onset of NC delamination from segmental plate levels of the axis where all cells are still epithelial. Various reasons might account for the latter observation, such as differences in the half-life of the two proteins, differential plasmid efficiency, and so on. On the other hand, we cannot rule out the possibility that the effects monitored with each of the mutants represent an underestimate of the true effect as a partial compensation by endogenous RhoA of the N19RhoB or GAP-RhoB effect (and *vice versa*) might have occurred. The observation that the effect of C3 transferase *in vivo *was somewhat more significant agrees with such a notion. Consistent with the involvement of both proteins in maintenance of the epithelial state, we observed that in the epithelial and flattening epithelioid progenitors, the distribution of both RhoA and RhoB was predominantly membrane-associated, a pattern reflecting the expression of post-translationally modified proteins in their active state [34]. Moreover, this pattern was gradually downregulated in cells about to delaminate and fully disappeared upon acquisition of a mesenchymal phenotype. Similar results were obtained upon direct visualization of active RhoGTPases with RBD-GST, which, together with results from the functional experiments, further strenghten the notion that active proteins relevant to NC EMT are membrane-associated. In addition to cell surface expression, RhoB-immunoreactive protein was expressed in the cytoplasm, likely in endosomal compartments [69] and this pattern did not change after NC cells delaminated, possibly reflecting the observed maintenance of *RhoB *mRNA and perhaps also of cytoplasmic RBD-GST binding in early migrating progenitors. Hence, vesicular RhoB could have other functions, not necessarily related to NC delamination, both before as well as during cell migration. Enhanced and premature delamination of NC cells is also observed upon inhibition of Rock signaling. The latter, although classically considered to act downstream of RhoA, was recently found to mediate activities of RhoB in various contexts [70-73]; hence, membrane-associated RhoA and RhoB might signal through Rock to affect EMT of NC cells, whereas in endosomes, RhoB pimarily interacts with mDia to affect protein trafficking (see, for example, [74]) and yet unknown functions in the NC.

**Figure 11:**
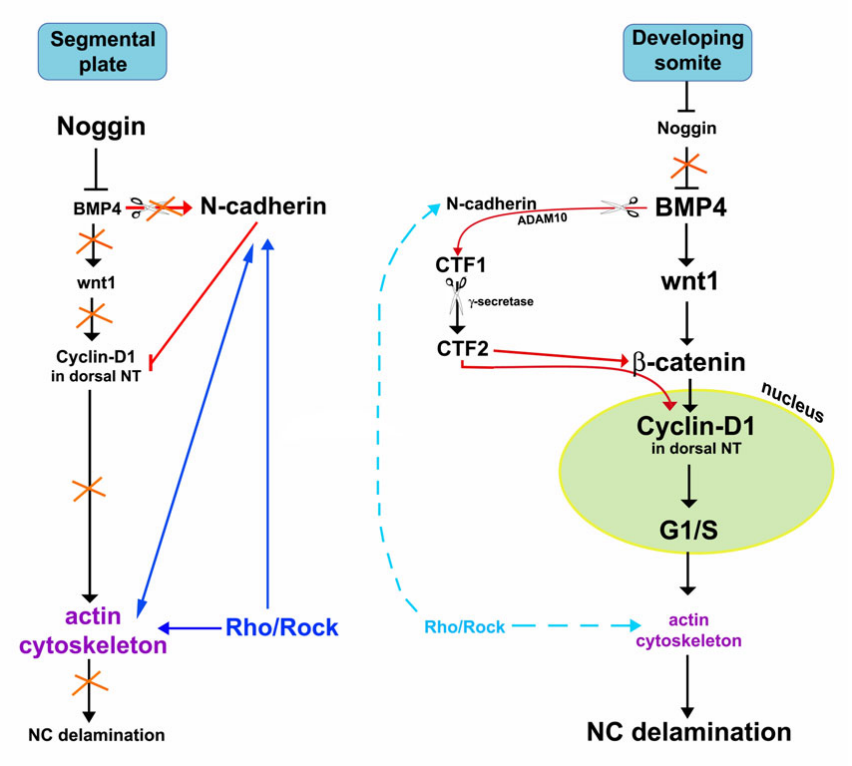
**The role of Rho/Rock signaling in the molecular network underlying neural crest (NC) delamination.** Opposite the segmental plate mesoderm, high levels of noggin result in low bone morphogenetic protein (BMP) activity, no Wnt1 transcription, low cyclin D1 in dorsal neural tube (NT) and no NC cells emigrating from the caudal NT. N-cadherin at this stage is expressed in the dorsal NT where it contributes to maintaining low cyclin D1 and lack of NC emigration. Rho activity through Rock helps maintaining membrane-bound N-cadherin and keeps a stable F-actin cytoskeleton. Together with the previous, direct N-cadherin-F-actin interactions contribute to the maintenance of epithelial premigratory NC. With ongoing development, opposite mature epithelial and dissociating somites, a factor emitted by the dorsomedial portion of the paraxial mesoderm inhibits noggin transcription in the NT, thereby relieving BMP activity. BMP4 in turn triggers Wnt1 transcription. Canonical Wnt signaling positively modulates transcription of cyclin D1, G1/S transition and NC cell delamination. In parallel, BMP4 via ADAM10 promotes N-cadherin cleavage into soluble CTF2. CTF2 may act in at least two ways, by upregulating β-catenin transcription and by binding β-catenin protein; we proposed that the complex translocates into the cell nucleus where transcription of target genes such as cyclin D1, followed by G1/S transition and epithelial-to-mesenchymal transition (EMT) of NC are stimulated. Hence, BMP activity transforms N-cadherin into a stimulatory signal (for details, see [31]. Concomitant with delamination, membrane-bound Rho proteins are downregulated, suggesting reduced activities of both Rho and Rock proteins; consequently, a dynamic turnover of stress fibers is made possible and N-cadherin association to the membrane is relieved. Altogether, these processes are compatible with generation of cellular movement downstream of the G1/S transition phase.

We show that the effects on NC emigration caused by changing the levels of Rho activity cannot be explained by altered cell proliferation, cell survival or cell specification. Thus, the enhanced cell emigration observed in Rho loss of function experiments may reflect premature depletion of the subset of transfected NC progenitors due to an earlier than normal loss of cell adhesion and cytoskeletal stability that characterize the epithelial state. Indeed, enhanced NC delamination produced by inhibiting Rho/Rock is accompanied by a substantial loss of actin stress fibers and focal adhesions. In addition, we demonstrate that membrane-bound N-cadherin is lost under these conditions, even if its normal proteolytic degradation is inhibited [31], and reciprocally, when preventing N-cadherin degradation, stable stress fibers, a representation of Rho activity, are kept. Furthermore, when endogenous Rho is activated by LPA, the observed inhibition of cell delamination is associated with maintenance of membrane N-cadherin *in ovo *and explants. This confirms that in the NC, Rho proteins together with N-cadherin are negative effectors of the generation of cellular movement. Consistent with our results, cooperation between cadherins and the Rho-dependent actin cytoskeleton were shown to control many aspects of epithelial biogenesis and maintenance [75-83]. On the one hand, cadherin-mediated adhesion is both necessary and sufficient for small GTPase activation, and on the other hand, sustained Rho activity is required for N-cadherin mediated adhesion, likely through maintenance of cytoskeletal stability [83,84]. Based on these observations, we propose that in the dorsal NT at premigratory levels of the axis, where membrane-associated N-cadherin is strongly expressed, Rho activity is maximal, thus contributing to preservation of the epithelial state of presumptive NC cells (Figure 11). When noggin activity is downregulated and BMP is consequently activated, N-cadherin is proteolytically degraded in the dorsal NT via a BMP and ADAM10-dependent mechanism [31]. Loss of membrane bound N-cadherin could signal a reduction in Rho activity via modifications of the actin cytoskeleton. Alternatively, or in addition, BMP, a key regulator of NC EMT, could contribute to RhoA degradation [64]. In this context, it is important to emphasize that BMP was shown to be necessary for inducing and maintaining transcription of *RhoB *mRNA in the dorsal NT [20,40]. However, monitoring mRNA expression is not a predictive factor for Rho activity as C3 transferase, despite abolishing Rho function, did not affect levels of *RhoB *mRNA (MG and CK, unpublished); hence, the regulation of *Rho *transcription and protein activity are separable events [85]. Additional levels of regulation should be considered as well; for instance, RhoB protein was shown to be stabilized against proteolytic degradation by transforming growth factor-β; in turn, RhoB antagonized transforming growth factor-β-dependent transcriptional activation [86]. Hence, the possibility should be considered that, in our system as well, stabilized RhoB antagonizes BMP-dependent EMT of NC cells.

Previously, we showed that G1/S transition is necessary for NC delamination [21]. Furthermore, this process is stimulated by BMP-dependent canonical Wnt signaling through stimulation of cyclin D1 transcription [14]. Loss or gain of Rho function did not alter G1/S transition in the NC system in spite of reported effects of Rho GTPases on cell cycle dynamics in other systems [87-92]. Nevertheless, for the first time, we observed that EMT of NC cells that were arrested in G1 by treatment with either mimosine or noggin can be rescued by inhibiting Rho or Rock activities. Under both conditions, the emigrating cells failed to incorporate BrdU, showing that G1/S transition can be dissociated from EMT. These data suggest that Rho/Rock act downstream of G1/S. Consistent with this interpretation and with positive and negative roles of cyclinD1 and Rho in NC EMT, respectively, it was shown that cyclin D1 triggers cellular migration in a model of cell metastasis through the inhibition of Rho GTP and RockII activity and signaling [93].

Although this study focused on the function on Rho signaling, the possibility that additional GTPases like Rac and Cdc42 are involved in NC delamination awaits further testing. Antagonistic activities of Rho and Rac were reported [94], raising the option that downregulation of Rho would, among other events, activate Rac and favor NC EMT. Hence, our findings underscore the complexity of the process of NC delamination by unraveling the involvement of additional factors and functional interactions. Altogether, these establish a growing genetic network responsible for the generation of NC motility.

## Conclusion

We demonstrate that Rho/Rock activity is necessary and sufficient for maintaining NC progenitors in an epithelial state without affecting their state of specification. In addition, Rho activity acts downstream of BMP and G1/S transition, two essential events for achieving successful NC delamination. Acting as a downstream effector, Rho via Rock maintains stable F-actin stress fibers through which it is likely to preserve N-cadherin associated to the membrane of NC progenitors. Together, we suggest that Rho activity negatively modulates NC delamination by stabilizing the actin cytoskeleton and intercellular adhesions mediated by N-cadherin.

## Materials and methods

### Embryos

Fertile quail (*Coturnix coturnix japonica*) eggs from commercial sources were used.

### Expression vectors and electroporation

DNA expression vectors employed were: pCAGGS-AFP, which served as control [95]; YFP-C1-Lyn (from Tobias Meyer); the specific Rho targeting construct p190-rhoB-C (GAP-RhoB; from Yi Zheng [59]); dominant-negative RhoB and RhoA lacking GTPase activity (N19-RhoA and N19-RhoB; from George Prendergast [58]); the C3-like ADP-Rybosyltransferase (from Klaus Aktories [96]); and xNoggin [97]. DNAs were subcloned into pCAGGS and either fused in frame to a GFP-encoding sequence or co-electroporated with control GFP. Experimental details are available upon request. DNA (3–5 mg/ml) was microinjected into the lumen of the NT of 15–18 somite-stage embryos at the level of the segmental plate and/or two recently formed somites. A four parameter Pulse*Agile *square wave electroporator (PA-4000, Cyto Pulse Sciences, Inc. Maryland, USA) was used to deliver three groups of sequential pulses as follows: 3 × 18 V, 20 ms each; 3 × 26 V, 15 ms each; 3 × 18 V, 20 ms each. Embryos were reincubated for an additional 16 h, some followed by a 1 h pulse of BrdU (10 mM). Other electroporated embryos were reincubated for 2 h followed by explantation of isolated neural primordia (see below).

### Explants of neural primordia

Intact or electroporated neural primordia containing premigratory NC were excized from segmental plate levels of 16–20 somite-stage embryos and then explanted onto 8-well chamber slides (Lab-Tek, Nunc, Rochester, NY, USA) pre-coated with fibronectin (50 μg/ml; Sigma, St. Louis, Mo, USA) as described [21]. Culture medium consisted of CHO-S-SFM II (Gibco-BRL, Gaithesburg, MD, USA) to which membrane-permeable C3 (CT04, 1 μg/ml; Cytoskeleton, Inc. Denver, Co, USA), Y27632 (15 μM; Sigma), the amino-acid mimosine (600 μM; Calbiochem, San Diego, CA, USA), LPA (1 μg/ml, replaced once after 8 h in explants kept for a total of about 16 h; Cayman Chemical Co. Ann Arbor, Mi, USA), and the selective ADAM10 inhibitor GI254023X (12 μM) [98,99], BMP4 (100 ng/ml; R&D Minneapolis, MN, USA), or combinations of the above were added.

### Grafting of LPA-containing pluronic gel

Pluronic F-127 gel was prepared as previously described [100] and mixed with a concentration of 100 μg/ml LPA. Pluronic gel is liquid at low temperature but sets at room temperature, thus remaining stable over the site of application for several hours. Small pieces of control or LPA-containing gels were placed dorsal to NTs at the level of the segmental plate mesoderm and embryos were further incubated for 8 or 16 h.

### Tissue processing, immunocytochemistry and in situ hybridization

Embryos were fixed with 4% formaldehyde, embedded in paraffin wax and sectioned at 5 or 10 μm. Rabbit anti-GFP (Molecular Probes, Eugene, OR, USA) was used at 1:500 in combination with HNK-1 or BrdU immunolabeling or combined with *in situ *hybridization for *FoxD3*, *Snail2 or Sox9 *[22-24]. Antibodies against intracellular or extracellular domains of N-cadherin were applied following methanol fixation as described [31]. Vinculin antibodies were from the Hybridoma Bank. Filamentous actin was visualized with phalloidin. RhoA was visualized with two different antibodies: 26C4 (monoclonal SC418; Santa Cruz Biotechnology, Santa Cruz, CA, USA), or rat monoclonal Lulu51 [101]. Likewise, RhoB was evidenced with polyclonal antibody 119 (SC-180; Santa Cruz Biotechnology) or with an anti-RhoB monoclonal antibody from the Hybridoma Bank [40]. All antibodies were found to specifically recognize their respective antigens [40,101]. For visualization of Rho proteins, explants were fixed in 10% trichloroacetic acid as previously described [101]. The active, GTP-bound form of Rho was localized using GST-Rho-binding domain of Rhotekin (RBD-GST, Cytoskeleton, Inc.) as previously described [102]. Nuclei were visualized with Hoechst. Embryo sections and explants were photographed using a DP70 (Olympus) cooled CCD digital camera mounted on a BX51 microscope (Olympus).

### Data analysis

NC delamination was monitored in at least 5 embryos per treatment out of 8–21 embryos showing a similar phenotype, as described [14]. Briefly, the number of GFP+ or Hoechst+ cells located up to the migration staging area was measured in 25 sections of control versus experimental hemi-NTs, and expressed as mean ± standard deviation of total cases monitored, respectively. The number of NC cells with mesenchymal morphology that exited explanted NTs was counted in 20–25 microscopic fields/explant, each comprising an area of 2,500 μm^2^. BrdU incorporation was measured as previously described [21]. Results represent the average number of cells per explant (± standard deviation of 5–6 cultures counted out of at least 12–16 cultures/treatment showing a similar phenotype) normalized to the length of the NT fragment. Significance of results was determined using the unpaired Student's *t*-test.

## Abbreviations

BMP: bone morphogenetic protein; BrdU: bromo-deoxyuridine; EMT: epithelial-to-mesenchymal transition; GFP: green fluorescent protein; LPA: lysophosphatidic acid; NC: neural crest; NT: neural tube; RBD-GST: Rho-binding domain of Rhotekin fused to glutathione S-transferase.

## Competing interests

The authors declare that they have no competing interests.

## Authors' contributions

MG And IS carried out experiments, analyzed the data, prepared the figures and participated in writing the manuscript. CK conceived the study, assisted in data analysis and wrote the manuscript.

## Supplementary Material

Additional file 1 **Inhibition of Rock signaling promotes premature neural crest delamination.** (A,B) Control. (C,D) Explants treated with Y27632. No neural crest (NC) delamination is apparent in controls 2 h following seeding (A) whereas many mesenchymal (M) cells already delaminated from treated neural tubes (NTs) (C). (B,D) The same explants 20 h following explantation. A flattened epithelioid (Ep) sheet separates in the control explant between the NT and the mesenchymal NC (B). In contrast, Y27632 enhanced NC delamination (note lower magnification in (D) when compared to (B) to include a greater number of cells) with no intermediate Ep pattern. Bar: 45 μM (A,B,C); 90 μM (D). Click here for file

Additional file 2 **Loss of F-actin in neural crest cells that received N19-rhoA or N19-rhoB.** Neural tubes were electroporated *in ovo *with control green fluorescent protein (GFP) (A-C), N19-rhoA (D-F) or N19-rhoB (G-I). Neural primordia were then isolated and explanted. In all cases, transfected progenitors delaminated from neural tube (NTs) (A,A',D,D',G,G'). Control GFP+ neural crest (NC) cells exhibited actin+ stress fibers (arrows in (B,C)) but NC cells that received either N19-rhoA or N19-rhoB were devoid of stress fibers (arrows in (E,F) and (H,I)) when compared both to control-GFP and to untransfected cells in the same cultures. Some adopted irregular morphologies were also observed upon C3 and Y27632 treatments (E and inset). (A,D,G) Phase contrast. (A',B,D',E,G'H) GFP immunostaining. (C,F,I) Phalloidin. M, mesenchymal. Bar: 70 μM (A,A',D,D',G,G'); 5 μM (B,C,E,F,H,I). Click here for file

Additional file 3 **Modulation of the F-actin cytoskeleton by Rho/Rock and N-cadherin in association with neural crest delamination.** Phalloidin staining of control explants (A) or of explants treated with Y27632 (B), lysophosphatidic acid (LPA) (C), GI254023X (E) and combinations of LPA+Y27632 (D) or GI254023X+Y27632 (F). Y27632 abrogated stress fibers normally seen under control conditions. In contrast, LPA and GI254023X strongly enhanced them while maintaining neural crest (NC) cells in an epithelial state. Both effects of LPA and GI254023X were reverted by co-treatment with Y27632 (see low magnification insets in (C,D)). Bar: 4.5 μM (A-F); 60 μM, insets in (C,D). Click here for file

Additional file 4 **Rho/Rock signaling modulate formation of vinculin-containing focal contacts.** Vinculin immunostaining of (A) focal attachment sites in control neural crest (NC) cells. (B) Y27632 strongly reduces the number of vinculin+ focal attachments in association with enhanced NC delamination and altered cell morphologies. (C) Treatment with lysophosphatidic acid (LPA) enhances vinculin immunostaining (images taken at identical conditions) and focal attachments in NC progenitors that failed to delaminate. Bar: 3 μM. Click here for file
