# Supplementary figures and images for: A negative modulatory role for rho and rho-associated kinase signaling in delamination of neural crest cells
Source: Neural Dev. 2008 Oct 22;3:27. doi: 10.1186/1749-8104-3-27 (PMC2577655; doi:10.1186/1749-8104-3-27)

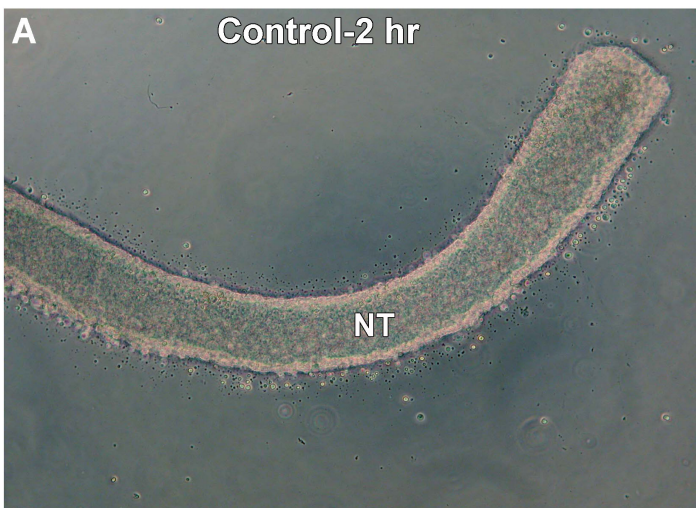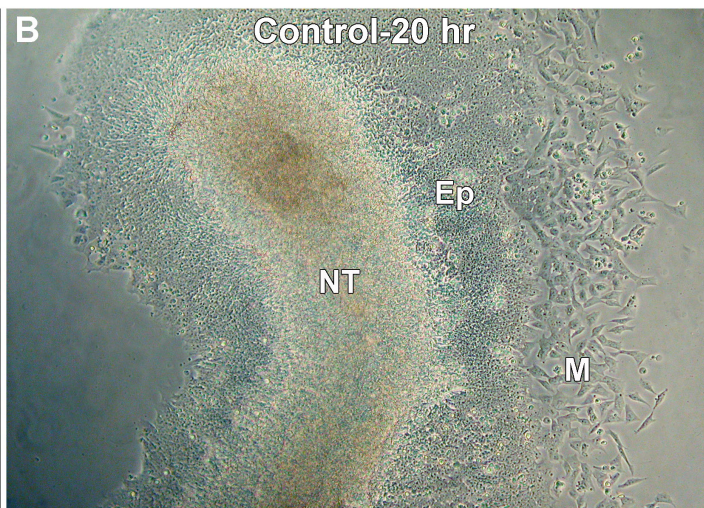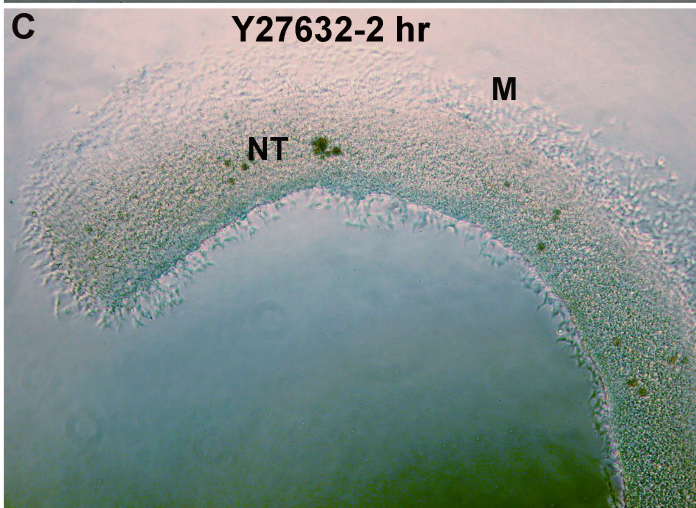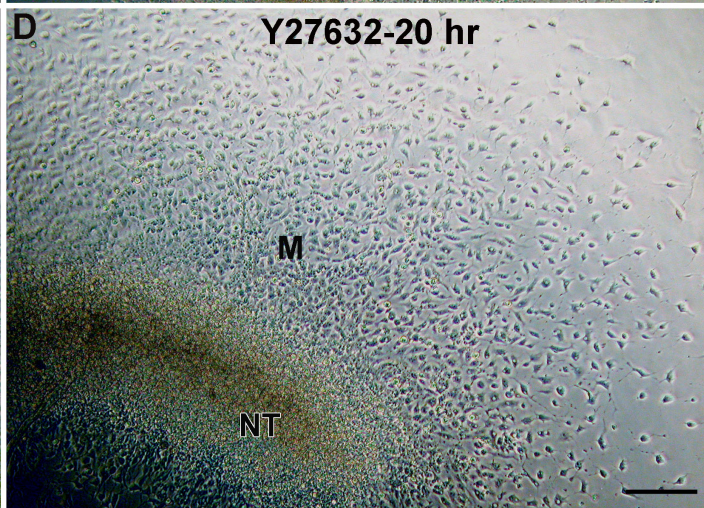

**Groysman et al- Additional Fig.1**

Supplement: Additional file 1 — Inhibition of Rock signaling promotes premature neural crest delamination. (A,B) Control. (C,D) Explants treated with Y27632. No neural crest (NC) delamination is apparent in controls 2 h following seeding (A) whereas many mesenchymal (M) cells already delaminated from treated neural tubes (NTs) (C). (B,D) The same explants 20 h following explantation. A flattened epithelioid (Ep) sheet separates in the control explant between the NT and the mesenchymal NC (B). In contrast, Y27632 enhanced NC delamination (note lower magnification in (D) when compared to (B) to include a greater number of cells) with no intermediate Ep pattern. Bar: 45 μM (A,B,C); 90 μM (D). [file 1749-8104-3-27-S1.pdf]

Control

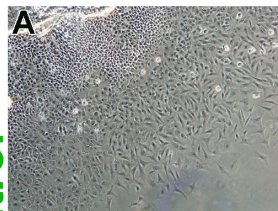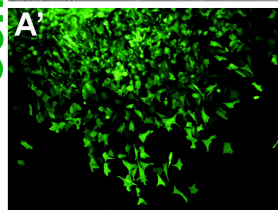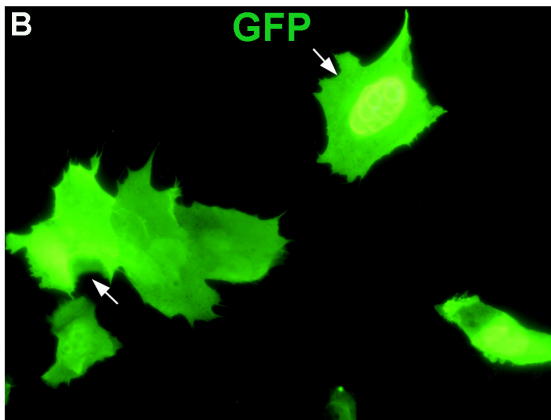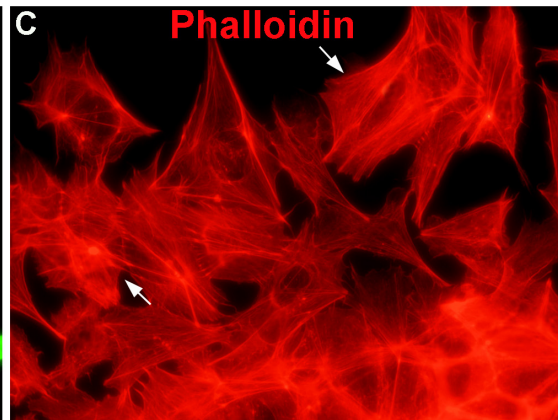

N19-RhoA

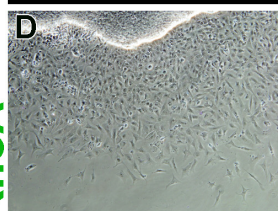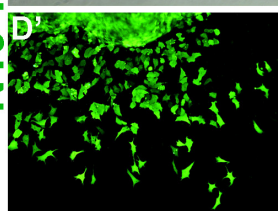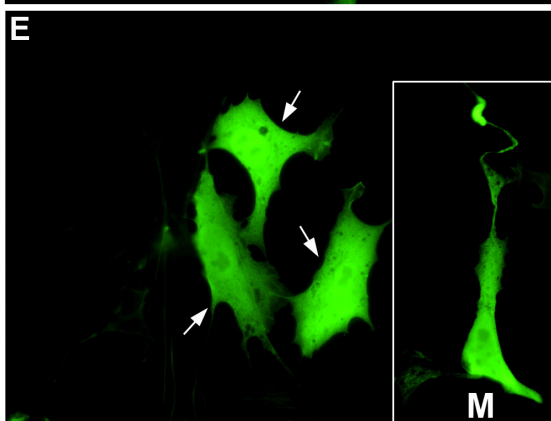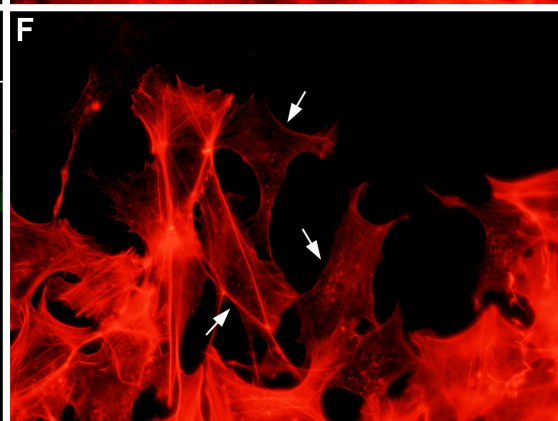

N19-RhoB

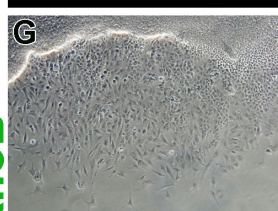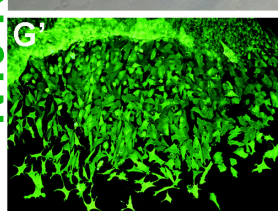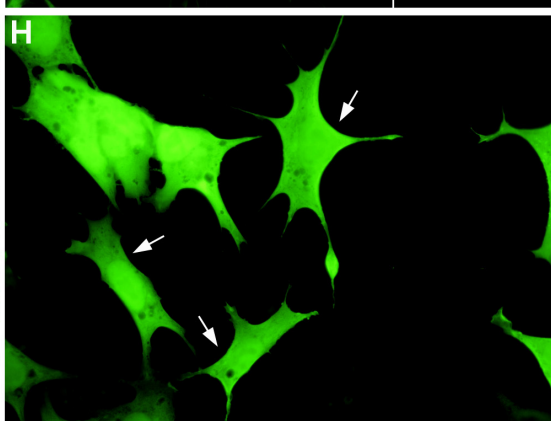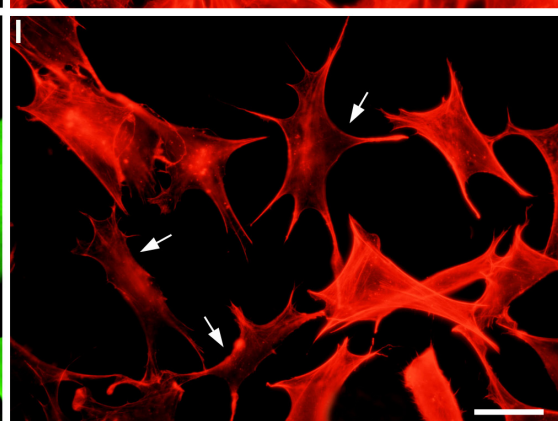

**Groysman et al- Additional Fig.2**

Supplement: Additional file 2 — Loss of F-actin in neural crest cells that received N19-rhoA or N19-rhoB. Neural tubes were electroporated in ovo with control green fluorescent protein (GFP) (A-C), N19-rhoA (D-F) or N19-rhoB (G-I). Neural primordia were then isolated and explanted. In all cases, transfected progenitors delaminated from neural tube (NTs) (A,A',D,D',G,G'). Control GFP+ neural crest (NC) cells exhibited actin+ stress fibers (arrows in (B,C)) but NC cells that received either N19-rhoA or N19-rhoB were devoid of stress fibers (arrows in (E,F) and (H,I)) when compared both to control-GFP and to untransfected cells in the same cultures. Some adopted irregular morphologies were also observed upon C3 and Y27632 treatments (E and inset). (A,D,G) Phase contrast. (A',B,D',E,G'H) GFP immunostaining. (C,F,I) Phalloidin. M, mesenchymal. Bar: 70 μM (A,A',D,D',G,G'); 5 μM (B,C,E,F,H,I). [file 1749-8104-3-27-S2.pdf]

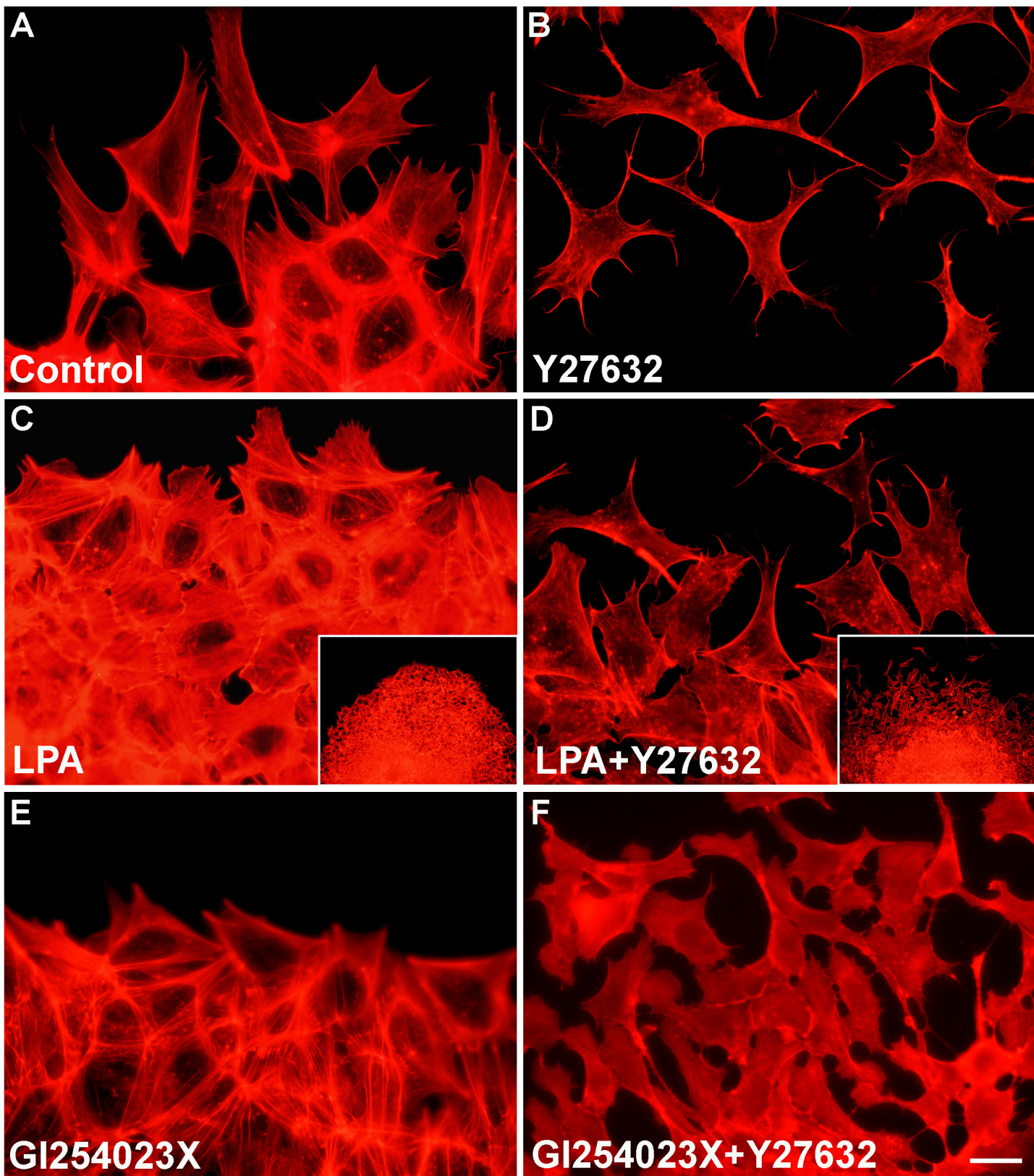

**Groysman et al- Additional Fig.3**

Supplement: Additional file 3 — Modulation of the F-actin cytoskeleton by Rho/Rock and N-cadherin in association with neural crest delamination. Phalloidin staining of control explants (A) or of explants treated with Y27632 (B), lysophosphatidic acid (LPA) (C), GI254023X (E) and combinations of LPA+Y27632 (D) or GI254023X+Y27632 (F). Y27632 abrogated stress fibers normally seen under control conditions. In contrast, LPA and GI254023X strongly enhanced them while maintaining neural crest (NC) cells in an epithelial state. Both effects of LPA and GI254023X were reverted by co-treatment with Y27632 (see low magnification insets in (C,D)). Bar: 4.5 μM (A-F); 60 μM, insets in (C,D). [file 1749-8104-3-27-S3.pdf]

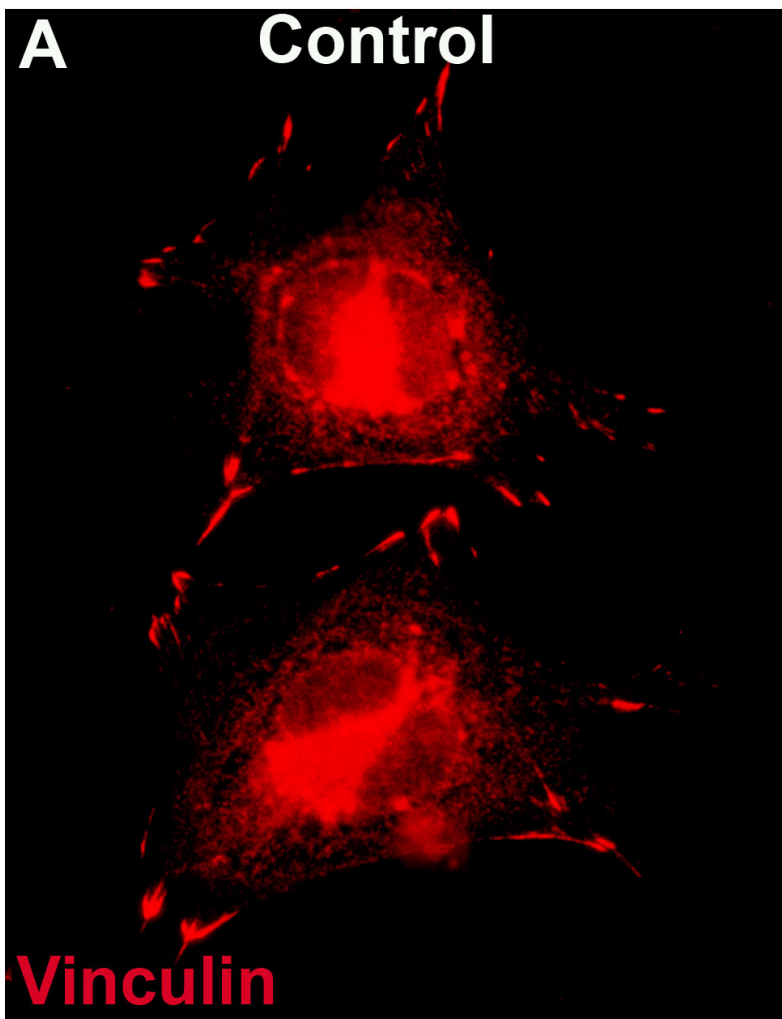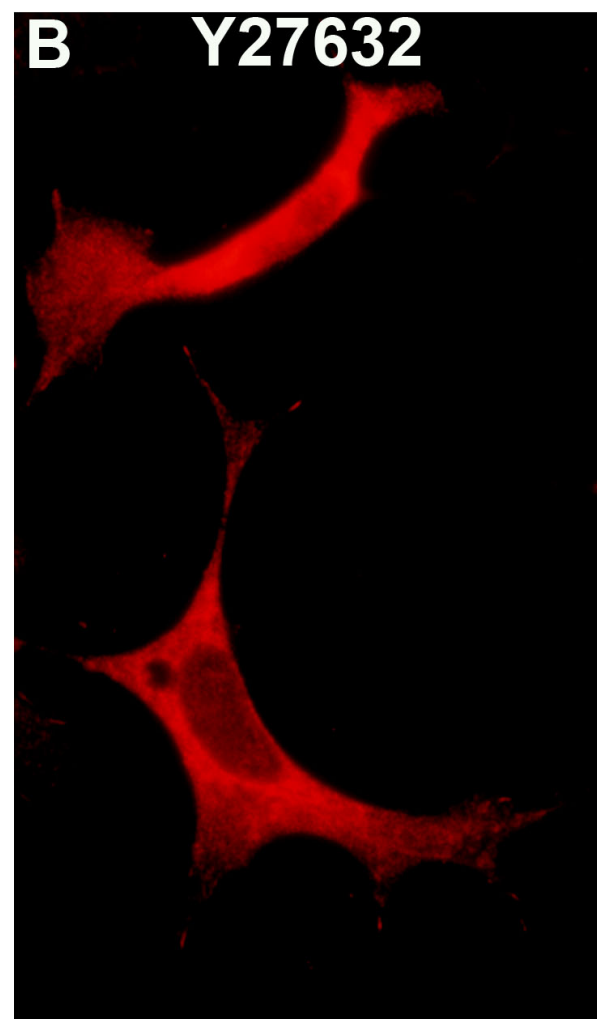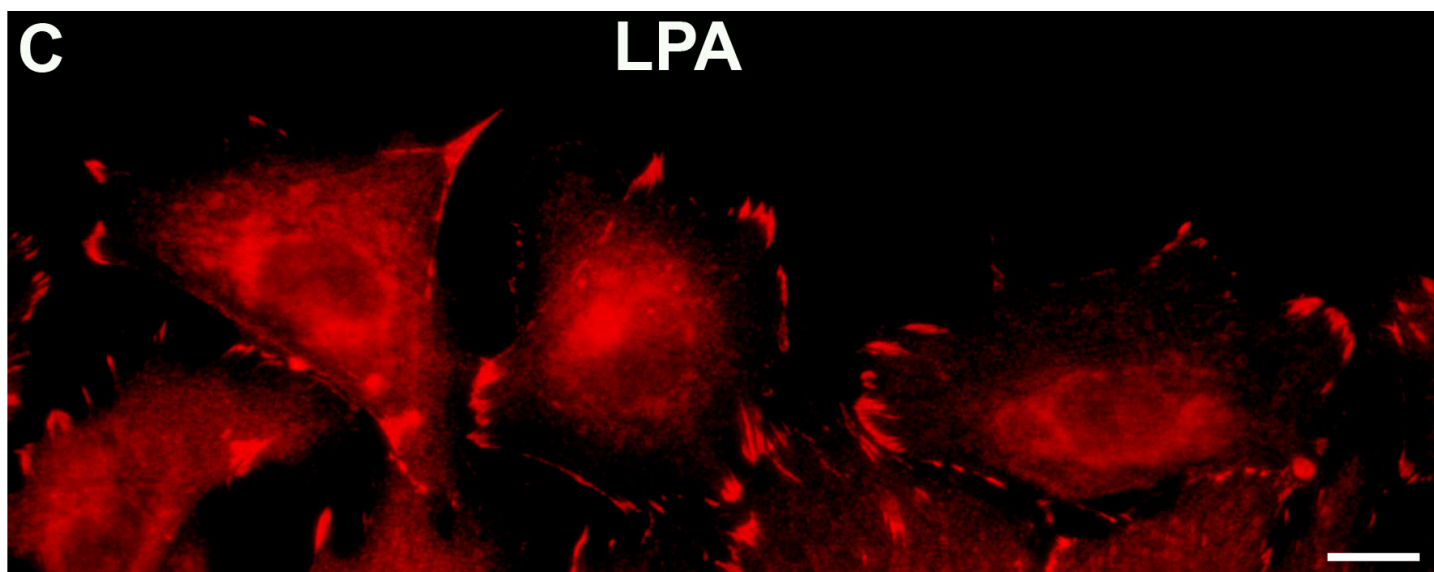

**Groysman et al- Additional Fig.4**

Supplement: Additional file 4 — Rho/Rock signaling modulate formation of vinculin-containing focal contacts. Vinculin immunostaining of (A) focal attachment sites in control neural crest (NC) cells. (B) Y27632 strongly reduces the number of vinculin+ focal attachments in association with enhanced NC delamination and altered cell morphologies. (C) Treatment with lysophosphatidic acid (LPA) enhances vinculin immunostaining (images taken at identical conditions) and focal attachments in NC progenitors that failed to delaminate. Bar: 3 μM. [file 1749-8104-3-27-S4.pdf]
